# Spatial Phenotype of the Mast Cell Population in Endometritis of Various Severities

**DOI:** 10.3390/cells15010038

**Published:** 2025-12-24

**Authors:** Sergey Mikhalev, Andrey Kostin, Mark Kurtser, Victor Radzinsky, Mekan Orazov, Alexander Alekhnovich, Aleksandra Prikhodko, Grigory Demyashkin, Ilya Klabukov, Denis Baranovskii, Daniel Elieh-Ali-Komi, Igor Buchwalow, Markus Tiemann, Liudmila Mikhaleva, Dmitrii Atiakshin

**Affiliations:** 1 N.I. Pirogov Russian National Research Medical University, 117997 Moscow, Russia; mikhalev@me.com (S.M.); m.kurtser@mcclinics.ru (M.K.); 2 RUDN University, 6 Miklukho-Maklaya St., 117198 Moscow, Russia; andocrey@mail.ru (A.K.); radzinsky@mail.ru (V.R.); omekan@mail.ru (M.O.); alekhnovich_av@pfur.ru (A.A.); prikhodko_at@pfur.ru (A.P.); buchwalow@pathologie-hh.de (I.B.); 3 I.M. Sechenov First Moscow State Medical University (Sechenov University), Trubetskaya St., 8/2, 119048 Moscow, Russia; dr.dga@mail.ru; 4 National Medical Research Radiological Center of the Ministry of Health of the Russian Federation, Koroleva St. 4, 249036 Obninsk, Russiadoc.baranovsky@gmail.com (D.B.); 5 Institute of Allergology, Charité-Universitätsmedizin Berlin, Corporate Member of Freie Universität Berlin and Humboldt-Universität Zu Berlin, 10117 Berlin, Germany; daniel.elieh-ali-komi@charite.de; 6 Fraunhofer Institute for Translational Medicine and Pharmacology ITMP, Immunology and Allergology, 12203 Berlin, Germany; 7 Institute for Hematopathology, Fangdieckstr. 75a, 22547 Hamburg, Germany; mtiemann@hp-hamburg.de; 8 Scientific Research Institute of Human Morphology Named After Academician A.P. Avtsyn, Russian Scientific Center of Surgery Named after Academician B.V. Petrovsky, 127051 Moscow, Russia; mikhalevalm@yandex.ru; 9 Research Institute of Experimental Biology and Medicine, Burdenko Voronezh State Medical University, 394036 Voronezh, Russia

**Keywords:** mast cells, tryptase, endometritis, spatial phenotyping, mapping, intercellular interactions, tissue microenvironment, immune landscape, fibrosis

## Abstract

Endometritis features an inflammatory milieu in the endometrium, accompanied by the recruitment of immunocompetent cells, including mast cells (MCs). The mechanisms underlying MC involvement in chronic endometritis (CE) and fibrous niche formation remain poorly understood, particularly regarding spatial intercellular interactions in situ. In this study, we used multiplex immunohistochemistry and quantitative immunofluorescence analysis to map the spatial phenotype of MC distribution. Standard histochemical techniques, monoplex and multiplex immunohistochemical staining technologies, light-field microscopy, epifluorescence, and confocal microscopy with multispectral imaging, combined with quantitative immunofluorescence analysis with AI application, were used to identify the spatial phenotyping of quantitative and qualitative features of the endometrial MC population in CE. The increased intensity of endometrial inflammation was accompanied by a rise in the profile of MC content in the endometrium; this accounted for a 0.014% increase in the control and 0.067%, 0.113%, and 0.206% increases in mild, moderate, and severe CE, respectively. We are the first to map the number of MCs that demonstrated loci of accumulations in the endometrium coinciding with foci of fibrous changes. The number of these foci correlated with the severity of chronic endometritis and the development of clinical signs. The frequency of juxtacrine and paracrine MC colocalization with other immunocompetent cells increased with increased CE activity and fibrotic changes: For CD8^+^ lymphocytes, colocalization increased from 4.6% in the control to 11.6%, 18.5%, and 28.0% in mild, moderate, and severe CE, respectively. For monocytes, colocalization increased from 5.6% in the control to 18.7%, 26.8%, and 28.8% in mild, moderate, and severe CE, respectively. For type 1 macrophages, colocalization increased from 5.6% in the control to 13.5%, 17.4%, and 24.6% in mild, moderate, and severe CE, respectively. For type 2 macrophages, colocalization increased from 3.4% in the control to 9.6%, 9.1%, and 21.5% in mild, moderate, and severe CE, respectively. Spatial patterns of juxtacrine and paracrine MC interactions with other immune cells may provide diagnostic algorithms for chronic endometritis, enabling targeted therapy and preventing fibrotic changes.

## 1. Introduction

Chronic endometritis (CE) is a persistent and subtle local inflammatory disease. Its incidence ranges from 15% to 57.5% in women with infertility, implantation failure during in vitro fertilization, or recurrent unexplained miscarriages [1]. Changes in the immune landscape of the reproductive system, apparently leading to abnormal intercellular interactions in the endometrial stroma, are considered to be an important cause of pregnancy termination [1,2]. However, the exact mechanisms regulating the architecture of the endometrial immune microenvironment under endometritis development have not yet been identified, even though most of the key physiological processes in the human reproductive tract, including follicle development, ovulation, implantation, pregnancy, childbirth, the postpartum period, remodeling, and menstruation, contain signs of an inflammatory component [1,3,4].

Mast cells (MCs), together with T lymphocytes, including natural killers, macrophages, and dendritic cells, are among the predominant immunocompetent cells of the reproductive system [5,6]. It is known that MCs are key regulatory elements of a specific tissue microenvironment and have unique capabilities in the implementation of innate and acquired immunity, as well as extracellular matrix remodeling [7,8]. With a wide repertoire of receptor apparatuses and secretory products, MCs are involved in maintaining both the canonical physiological parameters of local homeostasis and the initiation of inflammation, allergy, oncogenesis, fibrosis, and a number of other changes [9,10].

In specific tissue microenvironments, the role of mast cells (MCs) in the regulation of local homeostasis is crucial [11,12,13]. On the one hand, MCs express a wide range of receptors that provide highly sensitive mechanisms for the formation of a selective response to external and internal impact. On the other hand, MCs can selectively secrete various classes of mediators and alternative cytokine and chemokine profiles, thereby exerting a targeted effect on the immune and stromal landscapes of that particular tissue microenvironment [8]. MC tools are three main classes of mediators: preformed mediators, mediators of lipid origin and multiple cytokines, chemokines and growth factors formed after MC stimulation for the necessary modification of physiological reactions and immune functions [14,15]. MCs play a special role in the development of the pro-inflammatory background, regulating the state of numerous cells of the immune and stromal landscape, as well as the extracellular matrix of connective tissues [10,16,17,18,19,20,21,22,23].

Research on MC localization in the female reproductive system revealed different phenotypes depending on the content of specific proteases, with tryptase-positive MCs being most abundant in the myometrium compared to the basal and functional layers of the endometrium [24]. An increase in the endometrial MC population, along with the expression of tryptase mRNA, was found in recurrent miscarriage [25]. Some endometriotic lesions form a specific tissue microenvironment that attracts MCs, which, in turn, secrete proinflammatory mediators and contribute to chronic pelvic pain. It has been shown that MC recruitment can be promoted by stem cell factor (SCF), which is critical for mast cell expansion, differentiation, and survival for tissue, as well as the upregulation of VCAM1, CCL2, CCR1, and KITLG, which encodes stem cell factor (SCF), the ligand for the KIT receptor [26]. MCs can further act as an inducer of endometriosis development, maintenance of inflammation, and other effects, including late-onset preeclampsia [27,28,29].

As has been convincingly demonstrated, the local tissue microenvironment is significant for maintaining inflammation and fibrotic changes in the endometrium [30,31]. While the increasing number of intraorgan MCs in loci with fibrotic changes is known, we still lack specific details of their integration into the pathogenetic links of the disease [32,33]. The evidence that tryptase can lead to ovarian fibrosis through RNF152-mediated stabilization of B-cell lymphoma-extra-large (Bcl-xL, an inhibitor of apoptosis that acts through preventing cytochrome c release) is of great interest [34]. The number of MCs also correlated with the diagnostic markers of endometriosis, aquaporin 1, and the binding protein ZW10 [35]. Moreover, the lack of adequate standardization of individual immune cell counts is emphasized, as is the lack of consensus on what constitutes an abnormal level and its impact on implantation [36].

Current data remain fragmentary, limiting understanding of how the MC secretome, particularly tryptase, contributes to endometritis pathology. In this study, multiplex immunohistochemistry and quantitative immunofluorescence analysis technologies were used to map the spatial phenotype of MC distribution in the endometrium and to identify patterns of MC interaction with other immunocompetent cells in endometritis of various severities.

## 2. Materials and Methods

### 2.1. Samples and Preparation

This study was conducted with the approval of the local ethics committee of N.I. Pirogov Russian National Research Medical University, protocol No. 252, dated 25 June 2025. A total of 53 women with reproductive-age abnormal uterine bleeding (AUB) and a history of recurrent pregnancy loss (RPL) were included, all with histologically confirmed chronic endometritis. For comparison, a control group of 10 women with a regular menstrual cycle, intact reproductive function, and an endometrial assessment showing no abnormalities was selected, before they underwent surgery for uterine fibroids.

According to current SOPs, 5 μm paraffin-embedded sections fixed in 4% buffered formaldehyde were used for histochemical staining, while 2.5 μm sections were prepared for monoplex and multiplex immunohistochemistry [37].

Patients of reproductive age with pathologically and immunohistochemically verified CE were included. The exclusion criteria encompassed patients with reproductive system malignancies, current pregnancy or lactation, allergic reactions, severe systemic diseases (including diabetes mellitus and immunodeficiency disorders), and a history of cesarean section or myomectomy.

Endometrial samples were obtained via pipelle biopsy using a Pipelle vacuum syringe during the mid-proliferative phase (days 7–10 of the menstrual cycle). The biopsies underwent pathomorphological analysis following modified Noyes criteria for CE confirmation.

Based on the CE severity, participants were categorized into three groups: mild inflammation (*n* = 18), moderate inflammation (*n* = 16), and severe inflammation (*n* = 19). All participants provided written informed consent in line with the World Medical Association’s Declaration of Helsinki.

CE severity was classified based on histological staining and morphometric analysis: Mild CE was defined as no fibrosis, an arteriolar lumen area of 150–449 µm^2^ and diameter of 10.0–19.9 µm, with ≤1 CD138^+^ plasma cell. Moderate CE was defined as the presence of fibrosis, an arteriolar lumen area of 100–149 µm^2^ and diameter 8.0–9.9 µm, with 2–3 CD138^+^ plasma cells. Severe CE was defined as the presence of fibrosis, an arteriolar lumen area ≤ 99 µm^2^ and diameter ≤ 7.9 µm, with ≥4 CD138^+^ plasma cells [38].

For the IHC, we subjected the deparaffinized sections to antigen retrieval by heating the sections in a steamer with R-UNIVERSAL Epitope Recovery Buffer (Aptum Biologics Ltd., Southampton, SO16 8AD, UK) at 95 °C for 30 min. We omitted blocking the endogenous Fc receptors before incubation with primary antibodies, according to our earlier recommendations [39]. After antigen retrieval, quenching endogenous peroxidase when required, the sections were immunoreacted with primary antibodies. The list of primary antibodies used in this study is presented in Table 1. The immunohistochemical visualization of bound primary antibodies was performed manually, according to the standard protocol [37]. For manually performed immunostaining, primary antibodies were incubated overnight at +4 °C in an optimal dilution.

### 2.2. Primary and Secondary Antibodies

Bound primary antibodies were visualized using secondary antibodies (purchased from Dianova, Hamburg, Germany, and Molecular Probes, Darmstadt, Germany) conjugated with HRP. The list of secondary antibodies and other reagents used in this study is presented in Table 2. The nuclei were counterstained with Mayer’s hematoxylin; then, the sections were placed in a permanent mounting medium.

The bound primary antibodies were visualized using secondary antibodies (purchased from Dianova, Hamburg, Germany, and Molecular Probes, Darmstadt, Germany) conjugated with Alexa Fluor-488, Cy3, Alexa Fluor-555, Alexa Fluor-594, or Alexa Fluor-647 (Table 2 and Table 3). The final concentration of secondary antibodies was between 5 and 10 µg/mL PBS. Single and multiple immunofluorescence labeling were performed according to the standard protocols [37]. Sequential multiplex immunohistochemical staining for the simultaneous detection of tryptase, CD3, CD8, CD14, CD68, and CD163 (Table 2 and Table 3) was performed following Akoya Biosciences’ (Marlborough, MA, USA) recommendations on the use of OPAL series fluorochromes for the Mantra 2 Quantitative Pathology Imaging System (Table 2). In addition, when using OPAL series fluorochromes for repeated retrieval, the EZ-Retriever^®®^ System, MW015-IR (BioGenex, Fremont, CA, USA) was applied.

Since most MCs were tryptase-positive, we investigated the positivity of this protease to study MC colocalization with other cells. The designs of the diplex and multiplex immunohistochemical staining protocols are presented in Table 3.

### 2.3. Image Acquisition

For the study of microsections stained according to the histochemical and immunohistochemical protocols for light microscopy, the stained tissue sections were observed using a ZEISS AxioImager.Z2 equipped with a Zeiss alpha Plan-Apochromat objective 100×/1.46 OilDICM27, Zeiss Objective Plan-Apochromat 150×/1.35 GlycDICCorrM27, ZEISS Axiocam 712 color digital microscope camera, and ZEISS Axiocam 712 mono digital microscope camera (Carl Zeiss Vision, Jena, Germany). Photomicrographs were obtained in some cases with a Nikon D-Eclipse C1 Si confocal microscope based on a Nikon Eclipse 90i. The Mantra 2 Quantitative Pathology Imaging System for multiplex visualization (Akoya Biosciences, Marlborough, MA, USA) based on an Olympus BX43 microscope (Tokyo, Japan), equipped with a scientific-grade multispectral 12-bit monochrome high-sensitivity CCD camera with a liquid crystal tunable spectral filter, was used to determine the profile of specific proteases of mast cells and the immune and stromal landscape of the tumor microenvironment using the list of different OPAL fluorochromes.

Planimetric analysis was performed to identify the number of search cells (MCs, immune cells, and stromal cells) per unit area of the sections, as well as the absolute and relative number of MCs and other cells, elastic fibers using QuPath v0.5.1 software [40].

Mapping and imaging of the stained sections were performed after scanning the entire histological section using a digital pathology slide scanner (fluorescence) KF-FL-005 (Ningbo, Zhejiang, China), the ×40 objective of ScanScope CS (Leica Biosystems, Deer Park, IL, USA), and the Mantra 2 Quantitative Pathology Imaging System (Akoya Biosciences, Marlborough, MA, USA), based on an Olympus BX43 microscope.

QuPath version 0.5.1 software [40] was used to analyze images of the entire histological preparations. The Stardist extension was used to perform nuclear segmentation based on the DAPI signal [41]. The intensity threshold for the DAPI channel was defined with a pixel classifier built into Qupath, trained on expert annotations. Further classification of segmented detections by phenotype was achieved by training a neural network with point annotations and subsequent iterative verification by a specialist. Autofluorescence was minimized using a dedicated reagent kit and by optimizing exposure time during scanning. For cell colocalization analyses maximum distance for contacting cells was defined as the sum of the major semi-axes of the nuclei, averaged ≤10 μm. Distance categories for further analyses were defined as follows: <10 μm, contacting cells; 10–20 μm, cells within the paracrine interaction zone; >20 μm, non-interacting cells. The minimum Euclidean distance between the centroids of classified cells was calculated using the “Detection centroid distances” function built into QuPath. The resulting Euclidean distances were categorized to determine the frequency of direct cell contacts, paracrine interactions, and instances where cells were located outside the interaction zone. At each iteration, results were reviewed by a specialist and parameters adjusted as necessary. Quantitative data were exported from QuPath v0.5.1 to R for further visualization and analysis [42].

For every marker used in our immunofluorescence protocol, we conducted a parallel monochromatic IHC staining on sequential tissue sections. A direct comparison of the scanned whole-slide images from both methods demonstrated minimal discrepancy and high specificity of the antibody reactions, confirming that the IF signals accurately represent the target protein localization.

### 2.4. Controls

Control incubations included samples without primary antibodies or with substitution of the primary antibodies by the same IgG species (Dianova, Hamburg, Germany) at the same final concentration as the primary antibodies. Omission of either the primary or secondary antibody from the immunohistochemical reaction, as well as substitution with corresponding IgG at the same final concentration, resulted in the absence of immunostaining. The specific and selective staining of different cell types using primary antibodies from the same species within the same preparation was considered a sufficient control for immunostaining specificity. Representative isotype control images are provided in Supplementary Figure S29.

### 2.5. Statistics

Statistical analysis was performed using SPSS software (Version 13.0, IBM, Armonk, NY, USA). The results are presented as the mean (M) ± m (standard error of the mean). To assess the significance of the differences between the two groups, a Student’s *t*-test, or the Mann–Whitney U test in the case of a nonparametric distribution, was used. Significance was considered as * representing *p* < 0.05 compared to the control group and ** representing *p* < 0.01 compared to the control group. To increase the objectivity of the obtained statistical data and differences in the experimental groups, we also used the Bonferroni correction [43,44].

## 3. Results

### 3.1. MCs and Tryptase

**Figure 1:**
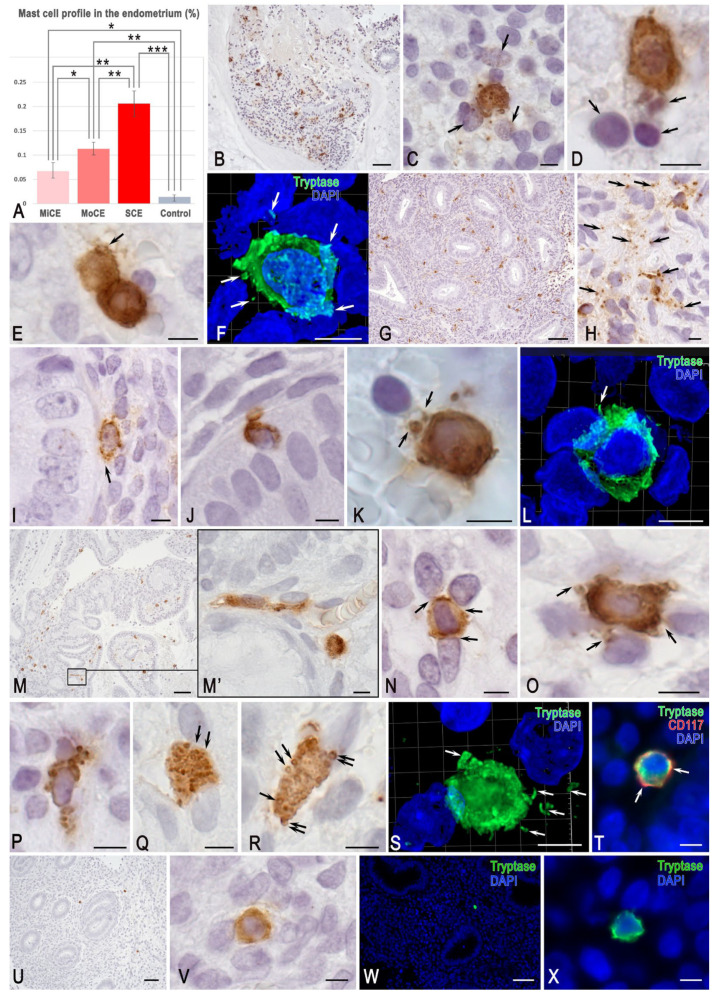
Mast cells in the endometrium. Technique: immunohistochemical tryptase detection. (**A**–**F**) **Mild chronic endometritis** (**A**). The mean relative abundance of MCs, expressed as a percentage of the total segmented cell count across groups. (**B**) Accumulation of MCs in a localized area of the endometrium. (**C**) An MC with signs of active tryptase secretion, which is visualized in some nuclei of endometrial cells (arrowed). (**D**) An MC colocalized with immunocompetent cells in the endothelial stroma (arrowed). (**E**) Interaction of two MCs (arrowed). (**F**) Active targeted tryptase secretion to tissue and cellular targets (arrowed). The 3D model is presented in Supplementary Figure S1. (**G**–**L**) **Moderate chronic endometritis**. (**G**) Accumulation of MCs in a limited locus of the uterine stromal lining. (**H**) Active tryptase secretion in the endometrial stroma with the formation of a large number of autonomous secretory granules located in the extracellular matrix (arrowed). (**I**) An MC adjacent to the basement membrane of the glandular epithelium (arrowed). (**J**) MC migration into the full-thick layer of the uterine gland epithelium. (K) Targeted tryptase secretion to a T lymphocyte (arrowed). (**L**) Simultaneous MC interaction with several cells of the stromal endometrium, including the formation of narrow cytoplasmic outgrowths (arrowed). The 3D model is presented in Supplementary Figure S2. (**M**–**S**) **Severe chronic endometritis**. (**M**) A locus of the increased MC content. (**M’**) An enlarged (**M**) fragment. (**N**,**O**) Juxtacrine MC interaction with several endometrial stromal cells and active tryptase secretion (arrowed). (**P**) MCs with high exosomal transport activity. (**Q**,**R**) MCs with large secretory granules; tryptase is localized in the peripheral region of these granules (arrowed). The point of secretory granule release into the extracellular matrix is indicated (double-arrowed). (**S**) Tryptase-positive cytoplasm without a nucleus with signs of active tryptase secretion (arrowed). The 3D model is presented in Supplementary Figure S3. (**T**) Preferential peripheral CD117 localization in MCs (arrowed). (**U**–**X**) **Control group**. Single mast cells in the endometrial stroma. Notes: *, **, and *** represent *p* < 0.05, 0.01, and 0.001 levels of significance difference, respectively. MiCE, mild chronic endometritis; MoCE, moderate chronic endometritis; and SCE, severe chronic endometritis. Scale: (**B**,**G**,**M**) at 50 μm, and the remaining images at 5 μm.

MCs in the uterine mucosa were mainly small-sized, up to 10–12 μm, with a round shape. Depending on the severity of endometritis, the number of MCs varied, with maximum values in patients with severe chronic endometritis and mild chronic endometritis (Figure 1A). In the control group, the MCs were most often located singly in the endometrial stroma, among other cells. However, in chronic endometritis, the distribution of MCs acquired histopographic features in the form of local accumulation within limited areas of the endometrium (Figure 1B,G,M). Uniform MC distribution was a rare phenomenon in the studied endometrial samples. Occasionally, MCs contacted each other (Figure 1E). MCs were often found near the endometrial glands, secreting tryptase to the basal membrane, and sometimes penetrating the layer of endothelial cells (Figure 1I,J). Forming loci of increased content, MCs were characterized by contacts with other stromal cells (Figure 1C,D,K,N–R). The increased severity of endometritis correlated with an increased area of loci with a high MC content. In addition, the secretory activity of MCs attracted attention. Due to this, the stroma could contain high levels of tryptase in the extracellular matrix, which ensured its high immunopositivity (Figure 1H). Large granules were rarely detected in endometrial MCs. Most frequently, tryptase was localized within immature type I and II secretory granules, which are poorly distinguishable by light microscopy, creating an impression of diffuse cytoplasmic staining (Figure 1C,D,F,L,N–P). The construction of 3D models clearly presents the intracellular cytotopography of tryptase, as well as the features of secretion (Supplementary Figures S1–S3). Sometimes, tryptase was localized in clearly visible secretory granules, especially at the periphery of the granules (Figure 1P–R). Secretory granules of certain MCs could be transported entirely into the extracellular matrix, and moments of such secretion were clearly visible (Figure 1P,R), although not visualized in all cells. In most MCs, the predominant mechanism of tryptase secretion degranulation was apparently manifested by exosomal secretion into a certain extracellular region; the structures of this region acquired diffuse immunopositivity to the specific protease (Figure 1K). In some cases, tryptase secretion was characterized by high activity resulting in the formation of large regions filled with MC granules or tryptase-positive cytoplasmic fragments (Figure 1C,O,S). In this case, MC granules contacted both endometrial cells and extracellular matrix structures. Sometimes, nuclei of other endometrial cells were positive for tryptase (Figure 1C). Almost all MCs in the endometrium were positive for CD117, regardless of the severity of endometritis, which was visualized mainly at the periphery of the cytoplasm (Figure 1T). Thus, immunodetection of this differentiation cluster may help to determine the size of the MC population in the endometrium.

### 3.2. MCs and CD8^+^ Cells

**Figure 2:**
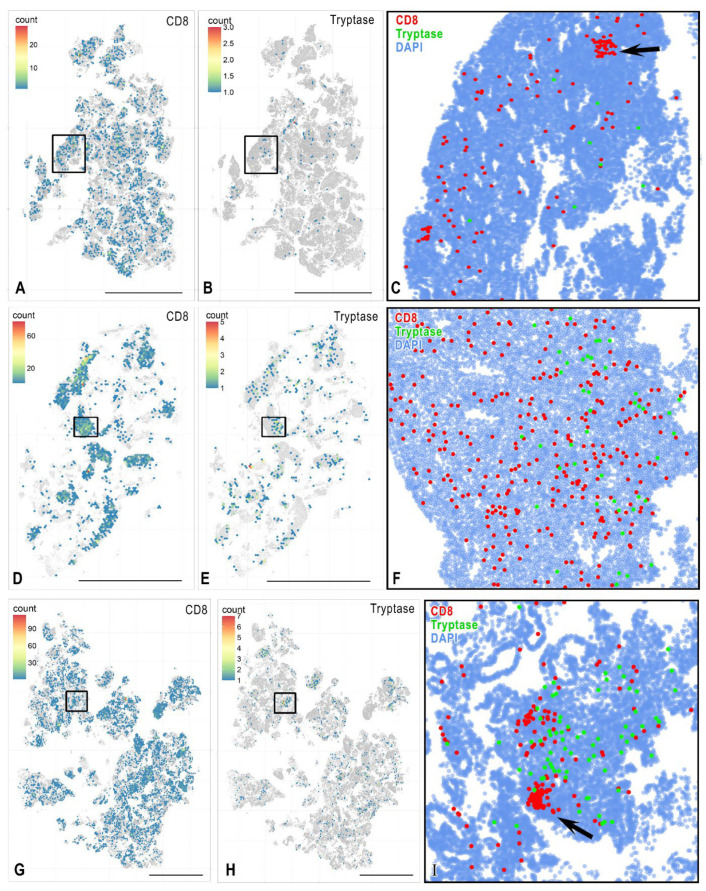
Histotopographic mapping of MCs and cytotoxic T lymphocytes in endometritis. (**A**–**C**) **Mild endometritis**. The heat scale demonstrates the geography of CD8^+^ T lymphocyte (**A**) and MC (**B**) levels in the endometrium, each colored hexagon represents an area of 8660 µm^2^. (**C**) Spatial phenotyping of MCs and killer T cells within the area bounded by the box in (**A**,**B**). The area of local accumulation of killer T cells is clearly visible (arrowed). (**D**–**F**) **Moderate endometritis**. The number of cytotoxic T lymphocytes (**D**) and MCs (**E**) in the endometrium are mapped, each colored hexagon represents an area of 8660 µm^2^. (**F**) Spatial phenotyping of MC tryptase and CD8^+^ lymphocytes within the area bounded by the box in (**D**,**E**). An increased number of MCs and their interaction with cytotoxic T lymphocytes becomes noticeable. (**G**–**I**) **Severe endometritis**. The highest numbers of MC and cytotoxic T lymphocytes in the endometrium are revealed by mapping the level of CD8 (**G**), tryptase (**H**), and the frequency of the mutual colocalization of immunocompetent cells (**I**), each colored hexagon represents an area of 8660 µm^2^. Areas of local accumulation of T-killers are visualized (arrowed). Scale: 5000 µm.

CD8^+^ cells formed certain “nests” in the endometrium, considered as loci with an increased content of cytotoxic lymphocytes (Figure 2A,C,G,I). These parameters varied in different patients; in particular, a more diffuse distribution of lymphocytes in the endometrium could be detected (Figure 2D,F).

**Figure 3:**
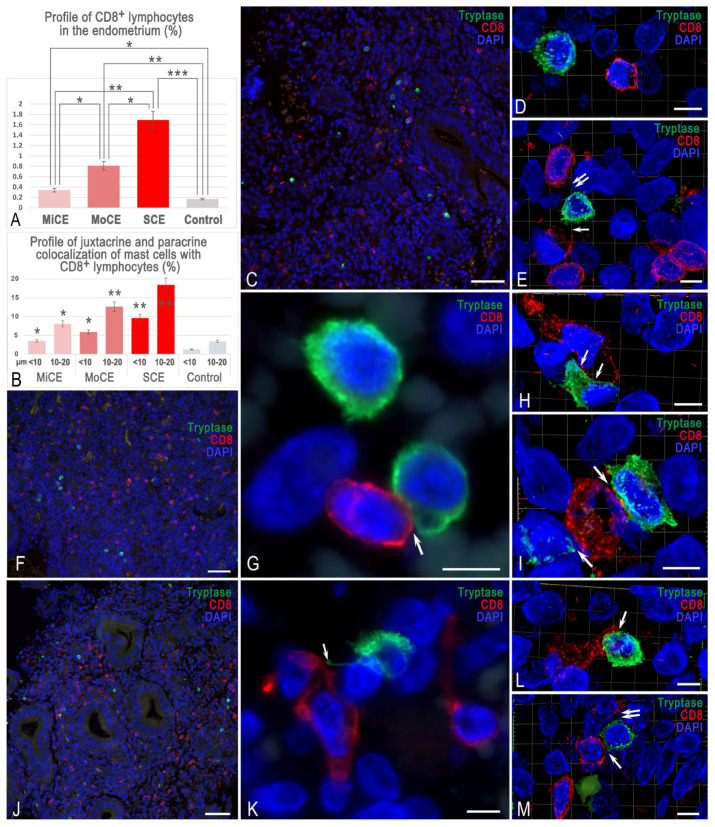
Specificity of the interaction of MCs with cytotoxic T lymphocytes in endometritis. Techniques: Diplex immunohistochemical staining of tryptase (green) and CD8 (red). Nuclei are contrasted with DAPI. (**A**) The mean relative abundance of cytotoxic lymphocytes, expressed as a percentage of the total segmented cell count across groups. The population of CD8^+^ lymphocytes increases with the severity of endometritis. (**B**) Frequency of contact events between MCs and CD8+ cytotoxic T lymphocytes at defined spatial distances. Values represent the percentage of the total MC population engaged in interactions at juxtacrine (<10 μm) or paracrine (10–20 μm) distances. The frequency of MC adhesion to killer T cells correlates with the severity of endometritis. (**C**–**E**) **Mild endometritis**. (**C**) MC localization in the loci of CD8^+^ lymphocyte accumulation. (**D**) Paracrine interaction of MCs and killer T cells. The 3D model is presented in Supplementary Figure S4. (**E**) Juxtacrine (arrowed) and paracrine (double arrowed) tryptase-mediated effects of MCs on cytotoxic T lymphocytes. The 3D model is presented in Supplementary Figure S5. (**F**–**I**) **Moderate endometritis**. (**F**) Elevated mast cell counts in the endometrial stroma are associated with a higher presence of CD8^+^ T-cells. (**G**) Two MCs, one of which is in close contact with a CD8^+^ lymphocyte (arrowed). (**H**) Juxtacrine colocalization of an MC and a cytotoxic T lymphocyte, forming a large contact area (arrowed). The 3D model is presented in Supplementary Figure S6. (**I**) Juxtacrine effects of two MCs on a killer T-cell (arrowed). The 3D image is presented in Supplementary Figure S7. (**J**–**M**) **Severe endometritis**. (**J**) Focal clustering of cytotoxic T lymphocytes coincides with a greater density of mast cells within specific tissue niches. (**K**) Targeted contact of MCs with a CD8^+^ lymphocyte via a thin cytoplasmic outgrowth (arrowed). (**L**) Close MC localization with a cytotoxic T lymphocyte (arrowed). The 3D image is presented in Supplementary Figure S8. (**M**) Juxtacrine (arrowed) and paracrine (double arrowed) effects of an MC on killer T cells. The 3D image is presented in Supplementary Figure S9. Notes: *, **, and *** represent *p* < 0.05, 0.01, and 0.001 levels of significance of difference compared to the control group, respectively. MiCE, mild chronic endometritis; MoCE, moderate chronic endometritis; and SCE, severe chronic endometritis. Scale: (**C**,**F**,**G**) at 50 μm, and the remaining images at 5 μm.

A local increase in lymphocytes in different endometrial areas was observed in the majority of patients (Figure 3A,C,G,I). The activity of interaction between MCs and cytotoxic T lymphocytes depended on the severity of endometritis and the number of CD8^+^ cells (Figure 3A,B). Mast cell density and CD8^+^ T-cell density per unit area were frequently increased together in the same regions (Figure 3C,F,J). Moreover, the increase in the severity of endometritis was accompanied by an increase in both the content of mast cells and CD8^+^ T cells per mm^2^ (Figure 1A, Figure 2, Figure 3A and Figure S30). Interestingly, the paracrine effects of MCs on lymphocytes were more frequent in mild endometritis (Figure 3B,D,E). The 3D modeling showed that MCs failed to contact lymphocytes most often in this group (Supplementary Figure S4). However, sometimes a single MC could exert both paracrine effects and juxtacrine effects (Figure 3E and Figure S5). The visualization of direct MC contacts with lymphocytes became more frequent with the increasing intensity of endometritis (Figure 2F,I and Figure 3G–I,L,M). The 3D modeling shows the formation of cell colocalization over a significant area (Supplementary Figures S6 and S7). The group with severe endometritis was characterized by the highest activity of MC interaction with T lymphocytes; this was histotopographically limited to small tissue niches in the endometrium.

The juxtacrine effect of MCs occurred in fairly significant areas of contact between cells, indicating the formation of potential immunological synapses, which can lead to other immunomodulatory effects. The volumetric modeling of such contacts supports mutual adaptive rearrangements of cell conformation, providing the most functional connection (Supplementary Figure S8). The discovery of MCs simultaneously affecting several T lymphocytes was also of great interest (Figure 3I,M and Figure S9). Moreover, in addition to the variants of immunocompetent cells interacting with each other under a close plasmalemma approach, other variants of cytotopographic changes in MCs appeared, particularly the formation of thin cytoplasmic outgrowths (Figure 3K).

We highlight the fairly frequent cases of MC interaction with CD8+ cells, which, in turn, contact other cells of the endometrial stroma (Figure 3G). Immunophenotyping of such cells in the future will allow us to identify new criteria for the development of the endometrium in the form of functional triads that promote the development of inflammation or fibrotic changes. In some cases, two MCs could be in the zone of juxtacrine influence on the same cytotoxic T lymphocyte (Figure 3I). Thus, the primary penetration of MCs into the endometrium is evidently due to immunological or microbiological reasons. This “settlement” of MCs entails a change in the composition of various components of the endometrial stroma that establish a pro-fibrotic tissue microenvironment. Furthermore, mast cells can recruit lymphocytes to these specific foci.

### 3.3. MCs and Monocytes

**Figure 4:**
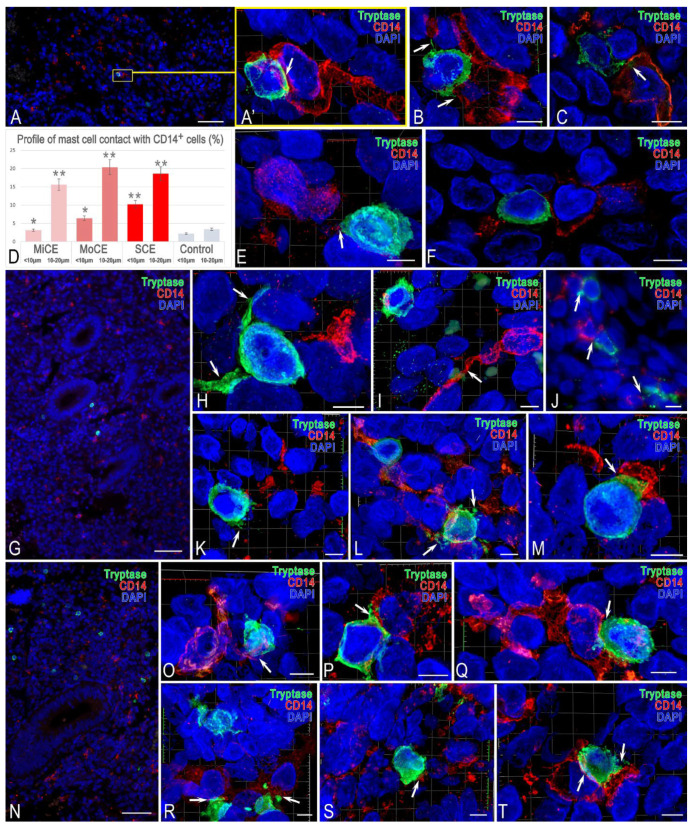
Features of MC interaction with CD14^+^ immunocompetent cells in endometritis of varying severity. (**A**–**F**) **Mild endometritis**. (**A**) General picture of monocytic cells prevailing over MCs in the endometrial stroma. (**A’**) Enlarged fragment of (**A**). A small MC is in close contact with a CD14^+^ cell with a pronounced cytoplasmic outgrowth (arrowed). The 3D model is presented in Supplementary Figure S10. (**B**,**C**). MC interaction of several monocytes with targeted tryptase secretion (arrowed). The 3D model (**B**) is presented in Supplementary Figure S11. (**D**) Frequency of contact events between MCs and monocytes (CD14+) at defined spatial distances. Values represent the percentage of the total MC population engaged in interactions at juxtacrine (<10 μm) or paracrine (10–20 μm) distances. An increased intensity of endometritis is accompanied by an increased frequency of immunocompetent cells’ colocalization, which is significant compared to the control group. (**E**) Tryptase secretion to a monocyte from a localized pole of an MC (arrowed). The 3D model is presented in Supplementary Figure S12. (**F**) A variant of MC interaction with several monocytes in the periglandular stroma. (**G**–**M**) **Moderate endometritis**. (**G**) An increase in monocytic cells in the endometrium. (**H**) Formation of cytoplasmic outgrowths of the MC (arrowed) and juxtacrine localization with a monocyte. The 3D model is presented in Supplementary Figure S13. (**I**) Paracrine MC localization with a CD14^+^ cell possessing an elongated outgrowth (arrowed). The 3D model (**B**) is presented in Supplementary Figure S14. (**J**) MC interaction with CD14^+^ cells within local tissue niches (arrowed). (**K**) Active tryptase secretion (arrowed). The 3D model is presented in Supplementary Figure S15. (**L**) Formation of a multicellular cluster of CD14^+^ and MCs interaction with active tryptase secretion (arrowed). The 3D model is presented in Supplementary Figure S16. (**M**) Close MC interaction of MCs with a monocyte (arrowed). The 3D model is presented in Supplementary Figure S17. (**N**–**T**) **Severe endometritis**. (**N**) Local increase in the number of MCs and monocyte population in the endometrium. (**O**) MC interaction with a CD14^+^ cell outgrowth (arrowed). The 3D model is presented in Supplementary Figure S18. (**P**) MC contacting a monocyte over a large area (arrowed). The 3D model is presented in Supplementary Figure S19. (**Q**–**T**) Various morphological variants of MC incorporation into the network of CD14^+^ endometrial cells (arrowed). The 3D models are presented in Supplementary Figures S20–S23. Notes: * and ** represent *p* < 0.05 and 0.01 compared to the control group, respectively. MiCE: mild chronic endometritis, MoCE: moderate chronic endometritis, SCE: severe chronic endometritis. Scale: (**A**,**G**,**N**) at 50 μm, with others at 5 μm.

MCs were characterized by a high degree of colocalization with CD14^+^ endometrial cells (Figure 4D). First, the juxtacrine and paracrine interaction with cells of the monocytic series also depended on the severity of endometritis (Figure 4A,G,N). In terms of cytotopography, contact with CD14^+^ cells occurred in different areas of the cytoplasm (Figure 4).

In some cases, this was contact with cytoplasmic outgrowths of MCs affecting small areas of monocytes (Figure 4E,F,K,O; Supplementary Figures S12, S15, and S18). However, sometimes the contact of MCs with monocytic cells occurred over a significant area of the cytoplasmic membrane, which was well visualized by confocal microscopy and the construction of 3D models (Figure 4A’–C,M,P,Q,T and Figures S10, S11, S17, S19, S20 and S23). The increasing severity of endometritis resulted in an increased area of MC contact with monocytic cells. Interestingly, CD14^+^ cells sometimes had an outgrowth-like and elongated shape, apparently forming contacts with several endometrial cells simultaneously (Figure 4H,I,L,O,Q,S and Figures S13, S14, S16, S18 and S22). In addition, MC integration into local clusters of CD14^+^ cells became noticeable in severe endometritis. Notably, there was an impression of a multicellular network formation consisting of contacting MCs and cells of the monocytic series; in some specific cases, they developed a functional syncytium.

### 3.4. MCs and Macrophages (M1 and M2)

**Figure 5:**
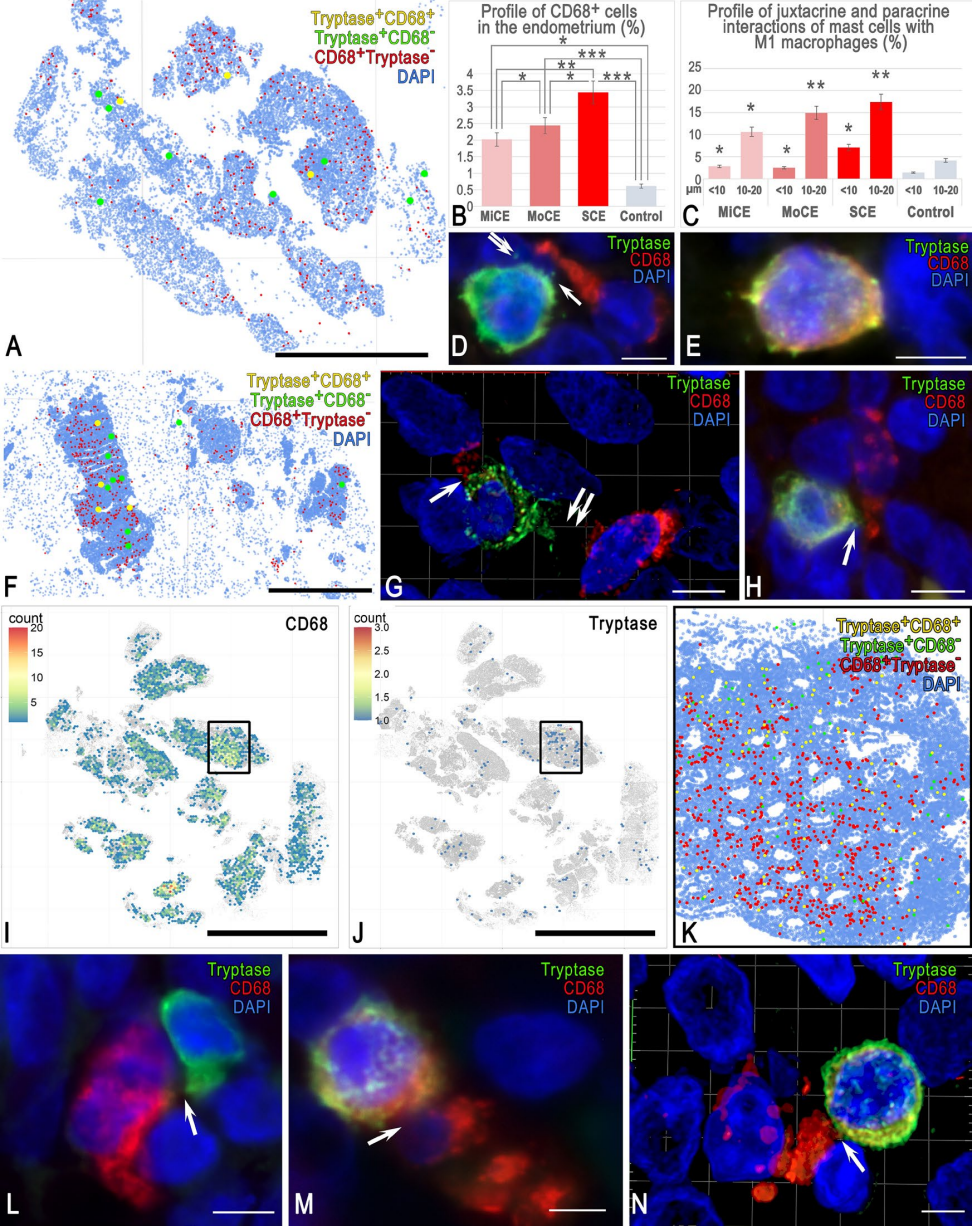
Analysis of CD68 and tryptase expression in MCs in the endometrium. (**A**–**E**) **Mild endometritis**. (**A**) Spatial phenotyping of CD68 and tryptase; CD68 is detected in some MCs. (**B**) The mean relative abundance of CD68^+^CD163^−^ macrophages, expressed as a percentage of the total segmented cell count across groups. (**C**) Frequency of contact events between MCs and CD68^+^CD163^−^ macrophages at defined spatial distances. Values represent the percentage of the total MC population engaged in interactions at juxtacrine (<10 μm) or paracrine (10–20 μm) distances. (**D**) Paracrine MCs and macrophages colocalization (arrowed), with tryptase in the nucleus of an adjacent cell (double arrowed). (**E**) MC with high CD68 content. (**F**–**H**) **Moderate endometritis**. (**F**) Spatial mapping of tryptase and CD68. The content of MCs increases, including those with CD68 expression. (**G**) Juxtacrine (arrowed) and paracrine interactions (double arrowed) of MCs with type 1 macrophages. The 3D model is presented in Supplementary Figure S24. (**H**) Targeted tryptase secretion to a type 1 macrophage (arrowed). (**I**–**N**) **Severe endometritis**. (**I**–**K**) Mapping of the intensity of CD68 (**I**) and tryptase-positive MCs (**J**) in the endometrium, each colored hexagon represents an area of 8660 µm^2^. (**K**) Spatial phenotyping of tryptase MCs and CD68 within the boxed area in (**I**,**J**). High content of type 1 macrophages and MCs, including those expressing CD68. More frequent colocalization of MCs and type 1 macrophages. (**K**) Targeting of a type 1 macrophage by an MC. (**M,N**) Juxtacrine localization of a CD68-rich MC to type 1 macrophages (arrowed). The 3D model is presented in Supplementary Figure S25. Notes: *, ** and *** represent *p* < 0.05, 0.01, and 0.001 compared to the control group, respectively. MiCE, mild chronic endometritis; MoCE, moderate chronic endometritis; and SCE, severe chronic endometritis. Scale: (**I**,**J**) at 5000 μm, (**A**,**F**) at 1000 μm, and the remaining images at 5 μm.

The study of mutual MC and type 1 macrophage colocalization demonstrated that MCs can contain a significant amount of macrosialin in the cytoplasm (Figure 5E). Diffuse distribution of CD68 was most often observed in MCs, although when clearly defined granules were present, it was located predominantly in the granules.

We found a correlation between macrosialin expression in MCs and the severity of endometritis. The largest pool of MCs immunopositive to CD68 was formed in severe endometritis. In addition, the profile of CD68^+^ cells in the total cohort of endometrial cells increased with the severity of endometritis (Figure 5B). The density of MC contacts with type 1 macrophages was maximal in the group with severe endometritis (Figure 5C). Interestingly, in the groups with mild and moderate endometritis, no significant difference in the frequency of MC and type 1 macrophage colocalization was observed. Spatial phenotyping demonstrated that the highest number of CD68^+^ macrophages in patients with moderate and severe chronic endometritis accumulated in areas containing MCs (Figure 5F,I,K). However, this pattern was not identified in some patients with mild chronic endometritis since MCs were practically absent or were present in insignificant quantities in the loci of macrophage accumulation (Figure 5A).

An interesting feature was that the 3D modeling revealed no targeted interactions of MCs with type 1 macrophages over a large area of the cytoplasm compared with monocytes (Figure 5G,N and Figures S24 and S25). Juxtacrine MC localization was accompanied by a locus of contact over a small area (Figure 5H,L,K).

**Figure 6:**
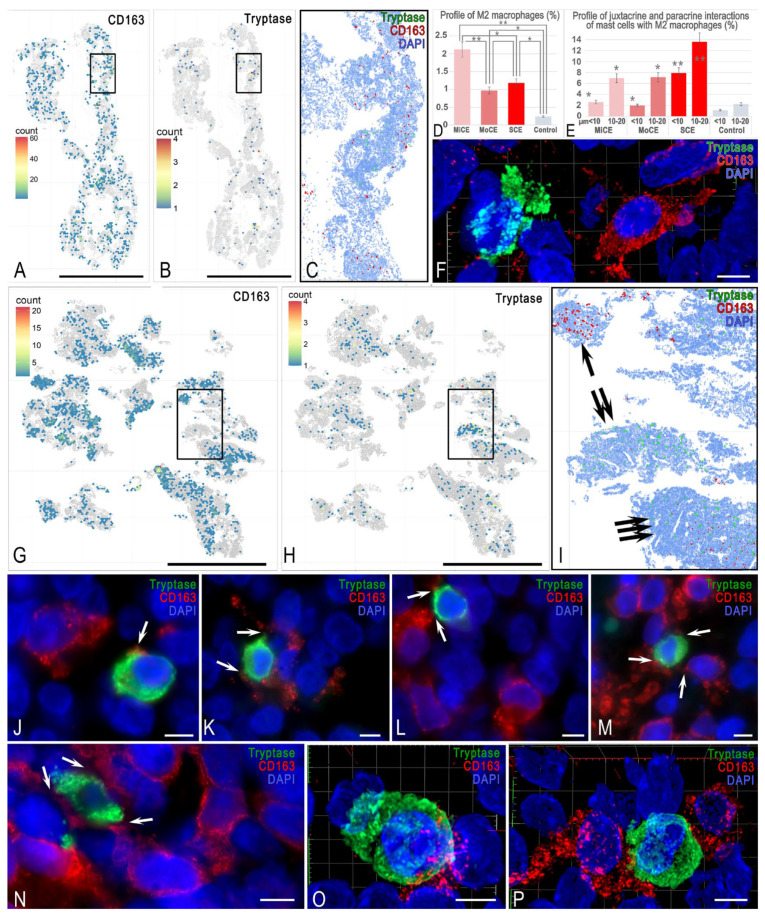
Features of the interaction between MCs and type 2 macrophages in the endometrium. (**A**–**F**) **Mild endometritis**. (**A**) Mapping the localization of CD163^+^ type 2 macrophages, with an uneven distribution of macrophages in the studied material, each colored hexagon represents an area of 8660 µm^2^. (**B**) Mapping the histoarchitecture of tryptase-positive MCs, where loci with high accumulation of MCs are detected each colored hexagon represents an area of 8660 µm^2^. (**C**) Mapping the mutual localization of MCs and type 2 macrophages within the area limited by the box in (**A**,**B**). (**D**) The mean relative abundance of CD163+ macrophages, expressed as a percentage of the total segmented cell count across groups. (**E**) Frequency of contact events between MCs and CD163+ macrophages at defined spatial distances. Values represent the percentage of the total MC population engaged in interactions at juxtacrine (<10 μm) or paracrine (10–20 μm) distances. Inflammation is accompanied by a significant increase in the frequency of MC contacts with type 2 macrophages. (**F**) An MC located at a paracrine distance from a type 2 macrophage. The 3D model is provided in Supplementary Figure S26. (**G**–**I**) **Moderate endometritis**. Spatial heterogeneity of the distribution of type 2 macrophages (**G**) and MCs (**H**) in the endometrium is clearly visible, each colored hexagon represents an area of 8660 µm^2^. (**I**) Spatial phenotyping of MCs and CD163^+^ macrophages in the area of the endometrium limited by the box in (**G**,**H**). Three variants of the spatial phenotype are observed: absence of macrophages in the area with accumulation of MCs (arrowed), absence of MCs in the area with accumulation of macrophages (double arrowed), and an area of colocalization of the two immunocompetent cells (triple arrowed). (**J**–**P**) **Severe endometritis**. (**J**) An MC at a paracrine distance from a type 2 macrophage. The contact locus of the CD163^+^ outgrowth on the MC surface is visible (arrowed). (**K**–**N**) Different patterns of juxtacrine interactions of MCs with type 2 macrophages (arrowed). Integration of MCs into the functional network of CD163^+^ macrophages is accompanied by simultaneous contact with two (**K**,**L**) and three (**M**,**N**) macrophages. (**O**,**P**) Three-dimensional images of juxtacrine interactions of MCs with type 2 macrophages. The large contact area is visible in the 3D models in Supplementary Figures S27 and S28. Notes: * and ** represent *p* < 0.01 and 0.001 compared to the control group, respectively. MiCE, mild chronic endometritis; MoCE, moderate chronic endometritis; and SCE, severe chronic endometritis. Scale: (**A**,**B**,**G**–**I**) at 5000 μm, (**C**) at 1000 μm, and the remaining images at 5 μm.

The study of MC interaction with type 2 macrophages revealed some specificity. First, the population profile of CD163^+^ macrophages was maximal in patients with mild chronic endometritis and decreased in patient groups with more severe endometritis (Figure 6D). However, mapping of MCs and type 2 macrophages in each group of the endometrium and in each group of patients revealed two subgroups.

In some patients, MCs were detected in the loci of type 2 macrophage accumulation, while in other patients, they were not. There were areas of the endometrium in which either MCs or type 2 macrophages were predominantly localized (Figure 6G–I).

However, there were areas with a high content of both MCs and type 2 macrophages, evidencing an active interaction of immunocompetent cells (Figure 6G–I). In this case, we could detect a kind of cellular network consisting of alternating MCs and CD163^+^ macrophages (Figure 6K).

Loci of active MC interaction with type 2 macrophages were observed in endometritis regardless of its severity. The paracrine effect of MCs on type 2 macrophages prevailed (Figure 6E and Figure S26). These histotopographic features had a high degree of specific variability. When MCs were embedded (integrated) into the type 2 macrophage network, they could contact two or more CD163^+^ cells (Figure 6K–N,P). In addition, cytoplasmic outgrowths of other cells with immunopositivity to CD163^+^ were often visualized on MCs (Figure 6J). The close contact area was clearly visualized when constructing 3D models of juxtacrine localization of these immunopositive cells (Supplementary Figures S27 and S28).

## 4. Discussion

Various mechanisms help MCs modulate the immune response, encompassing the enhancement or suppression of the development, survival, recruitment, activation, maturation, and proliferation of immune cells of both innate and adaptive immunity [45,46]. One of the preformed mediators with pronounced proinflammatory properties is tryptase [47,48,49,50,51]. Tryptase is the most abundant component of human MCs and as such acts as the specific protease that is the most convenient marker for detecting an entire MC population [21,33,52,53]. Therefore, the increase in tryptase-positive MCs in the uterine mucosa during the development of endometritis detected in this study indirectly supports the other previously reported results [25,26].

Tryptase is a direct participant in the formation of a proinflammatory background in the local tissue microenvironment. Spatial patterns of tryptase secretion during MC activation are the most important pathogenetic event for the development of inflammation, the pronounced intensity or chronic nature of which inevitably leads to the formation of profibrogenic niches with excessive formation of the extracellular matrix.

These loci of fibrous changes can merge with disease progression and capture larger areas of the endometrium. Chronic activation of MCs can induce persistent increased sensitivity to several components of the specific tissue microenvironment due to the restructuring of its own receptor apparatus, which provokes excessive secretion of biogenic amines, cytokines, and specific proteases with proinflammatory activity [54,55].

MC tryptase is characterized by high biological activity affecting the state of many cellular and non-cellular components of the tissue microenvironment [33,47,56,57]. A number of studies have shown close tryptase involvement in the processes of angiogenesis [58,59], combined with connective tissue remodeling and secretion of growth factors, cytokines, chemokines, and matrix metalloproteinases [60].

Tryptase potentiates the development of inflammation by interacting with Protease-Activated Receptor-2 (PAR-2) on various phenotypes of tissue microenvironment cells [60]. Tryptase is also known to cause a sustained increase in PAR-2 receptor expression in target cells, enhancing the effects of inflammation [58,61,62].

Recently, the epigenetic effects of tryptase on target cells have been widely discussed [63,64,65,66]. MCs interact with other cells directly, secreting mediators and releasing exosomes, which is critical in modulating immune responses through intercellular communication, influencing both innate and adaptive immunity [12].

Studies on the fundamental significance of tryptase transport into cell nuclei have shown the ability of the protease to process core histones in the N-terminal tail and alter transcription processes [63,65,66]. In this case, the enzymatic activity of tryptase in the nucleus is stabilized by DNA molecules, which allows for long-term regulation of the state of histones [63,66,67]. This principle of regulation of histone epigenetic modification in nuclei represents special functions of MC tryptase [66].

In our study, we first profiled the functional architecture of MC interaction with immunocompetent endometrial cells and demonstrated the key mechanisms of tryptase participation in the development of intraorgan inflammation. We have previously shown that varying degrees of chronic endometritis activity are regulated by a complex interaction of immune and cellular factors, including stromal and epithelial cells, building a complex system of intercellular communications [68]. The results suggest that with endometritis of various severities, MC interaction with other subpopulations of immune cells of the endometrial tissue microenvironment significantly changes, despite the personal features of each patient.

Thus, our results suppose the use of epigenetic mechanisms in the development of biological effects of tryptase in both extracrine and paracrine effects of MCs on monocytes, macrophages, lymphocytes, and other endometrial cells.

It is possible to assume direct MC participation in the macrophage polarization. Our results support the hypothesis that juxtacrine and paracrine effects of MCs on monocytes promote the transformation of monocytes into type 1 or 2 macrophages, thus creating a morphological basis for the proinflammatory or profibrotic status of the tissue microenvironment. Spatial phenotyping and profiling of the interaction intensity between MCs, monocytes, and macrophages indicate the ability of MCs to participate, for the required time interval, in organizing a specific architecture of functional intercellular networks of the endometrial immune landscape manifesting a syncytial nature.

This multicellular functional structure promotes the synchronization of the activity of immunocompetent cells in a certain locus for the implementation of specific morphogenetic effects, including proinflammatory and profibrotic changes. In particular, by binding type 2 macrophages to each other, MCs can coordinate their secretory potential and enhance fibrotic effects, creating tissue niches with a special status of an integrative–buffer metabolic environment in certain areas of the tissue microenvironment measuring 200 μm or more. In particular, a close correlation of CD14^+^ monocytes and CD163^+^ macrophages with the severity of fibrosis has been shown in other organs, including the liver [69].

Arguably, the conclusions about the different nature of the distribution of CD163^+^ cells in the form of single or grouped macrophages in the lumens of the superficial glands, mainly in the luteal phase [36], can be explained by the degree of cooperation with the MCs that we identified.

Based on our results, the data on the marked increase in the CD163-positivity of cells in the endometrial glandular compartment, along with CD19^+^ in case of implantation failures [70], can be supplemented by an analysis of MC expression. Accordingly, the use of a larger number of endometrial immune cells in the middle of the luteal phase of the menstrual cycle as predictors increases the accuracy of predicting the outcomes of assisted reproductive technologies [71].

Notably, the proportion of mast cells exhibiting macrosialin (CD68) expression was increased. The predominant location of macrosialin, or LAMP-4, in the cell is in late endosomes, where it functions in the transport of peptides or antigen processing and ensuring phagocytosis [72,73,74]. In addition, CD68 plays a key role in various physiological and pathological processes, including inflammation [75]. CD68 can quickly move between the plasma membrane and endosomes [76], causing the diffuse staining of the MC cytoplasm detected in our study, along with the accumulation of macrosialin in secretory granules. The increase in CD68 may indicate a more active MC participation in infection-induced endometritis in the processing or presentation of an antigen for subsequent immunogenesis. Our findings on the increase in the juxtacrine and paracrine interactions of MCs with CD8^+^ T lymphocytes suggest the participation of MCs in the activation of cytotoxic lymphocytes using perforin and granzyme. Our results are consistent with the known immunomodulatory effects of MCs, including the activation and proliferation of antigen-specific CD8^+^ T cells through direct immune contact with subsequent antigen presentation dependent on major histocompatibility complex class I [45].

Recent studies have shown a close involvement of CD8^+^ lymphocytes in the implementation of fibrotic changes in a number of organs [34,77]. Notably, CD8^+^ lymphocytes can acquire greater cytotoxicity due to the effect of MCs on T cells and the epigenetic effects of tryptase [64,66,67,78]. Thus, the increase in the CD8^+^ T cell population in the endometrium, on the one hand, may be a consequence of increased migration from the microcirculatory bed, due to more active MC interaction with endothelial cells. On the other hand, it is necessary to take into account that the targeted MC interaction with CD8^+^ lymphocytes is accompanied by an increase in cytotoxicity and their profibrotic effects. When comparing the results of this study with the detection of the fibrous extracellular matrix component, the loci in which active MC interaction with phagocytic cells and cytotoxic lymphocytes was detected contained a higher content of extracellular matrix. This conclusion was shown not only by comparing the results of silver impregnation, which allows the visualization of collagen and reticular fibers, but also via polarization microscopy data, after staining with picrosirius red, with the possibility of detecting collagen types I and III [68].

Thus, it is obvious that MCs are an important cause of inflammation and fibrosis at the level of the local tissue microenvironment; their scope can increase significantly with disease progression. It is necessary to take into account the ability of MCs to form certain clusters with the involvement of other cells in a temporarily formed functional syncytium, as demonstrated in our study; these can result in quite pronounced and rapid changes in the endometrial microenvironment over a short period of time. To gain a more detailed understanding of the biology of mast cells in the development of endometritis of various severities we recommended continuing research using co-indexing of transcriptomes and epitopes (CITE) to the spatial dimension [79], multimodal tri-omics mapping [80] and Spatially Resolved Panoramic in vivo CRISPR Screen via Perturb-DBiT [81].

Thus, for the first time, our study identified spatial patterns of mast cell integration into the immune landscape of the endometrium with the formation of multicomponent morphogenetic clusters that provide additional risks of inflammatory change progression and, as a consequence, remodeling of the fibrous extracellular matrix [82,83,84,85].

## 5. Conclusions

The patterns of juxtacrine and paracrine interactions of mast cells with other endometrial cell populations reveal new mechanisms of the targeted induction of inflammatory and fibrotic changes in the local tissue microenvironment. The discovered immunomodulatory mechanism of MCs goes beyond the biological effects of soluble mediators and includes spatial features of interaction with other cell populations of both innate and acquired endometrial immunity. Pharmacological correction of MC activity or individual components of the secretome is a promising direction for studying the risks of endometritis development and the potential for innovative diagnostics and effective prevention of the negative consequences of chronic endometritis.

## Figures and Tables

**Table 1:** Primary antibodies used in this study.

Antibody	Detecting Cell	Host	Catalog Nr.	Dilution	Source
Tryptase	Mast cells	Mouse monoclonal	#ab2378	1:4000	AbCam, Cambridge, UK
CD3	T lymphocytes	Rabbit monoclonal	ab16669	1:150	AbCam, Cambridge, UK
CD8	Cytotoxic T lymphocyte	Rabbit monoclonal	ab101500	1:100	AbCam, Cambridge, UK
CD14	Monocyte	Rabbit monoclonal	EPR3653	RTU *	Cell Marque, Rocklin, CA 95677, USA
CD20	B-lymphocytes	Rabbit monoclonal	ab166865	1:200	AbCam, Cambridge, UK
CD31	Endothelium	Rabbit monoclonal	ab182981	1:2000	AbCam, Cambridge, UK
CD68	Macrophages	Mouse monoclonal	ab955	1:3000	AbCam, Cambridge, UK
CD38	Plasma cells	Rabbit monoclonal	ab108403	1:500	AbCam, Cambridge, UK
CD163	Macrophage type 2	Rabbit monoclonal	ab182422	1:500	AbCam, Cambridge, UK

* RTU: ready to use.

**Table 2:** Secondary antibodies and other reagents.

Antibodies and Other Reagents	Source	Dilution	Label
Goat Anti-Mouse IgG Ab (#ab97035)	AbCam, Cambridge, UK	1/300	Cy3
Goat Anti-Rabbit IgG Ab (#ab150077)	AbCam, Cambridge, UK	1/300	Alexa Fluor 488
Goat Anti-Rabbit IgG H&L (Alexa Fluor^®®^ 555) (#ab150078)	AbCam, Cambridge, UK	1/300	Alexa Fluor 555
Goat Anti-Mouse IgG H&L (Alexa Fluor^®®^ 555) preadsorbed (#ab150118)	AbCam, Cambridge, UK	1/300	Alexa Fluor 555
Goat Anti-Mouse IgG H&L (Alexa Fluor^®®^ 594) (#ab150116)	AbCam, Cambridge, UK	1/200	Alexa Fluor 594
Goat Anti-Mouse IgG H&L (Alexa Fluor^®®^ 647) (#ab150115)	AbCam, Cambridge, UK	1/200	Alexa Fluor 647
Goat Anti-Rabbit IgG H&L (Alexa Fluor^®®^ 647) (#ab150079)	AbCam, Cambridge, UK	1/200	Alexa Fluor 647
DAPI FP1501001KT	Akoya Biosciences, Marlborough, MA, USA	RTU *	DAPI
1X Plus Auto Amplification Diluent FP1609	Akoya Biosciences, Marlborough, MA, USA	RTU	w/o
Opal Polymer HRP Ms + Rb Akoya Biosciences ARH1001EA	Akoya Biosciences, Marlborough, MA, USA	RTU	HRP
Antibody Diluent/Block buffer Akoya Biosciences ARD1001EA	Akoya Biosciences, Marlborough, MA, USA	RTU	w/o
Secondary antibodies conjugated with horseradish peroxidase FP1500001KT	Akoya Biosciences, Marlborough, MA, USA	RTU	Opal 480
Secondary antibodies conjugated with horseradish peroxidase Opal 540 Reagent (#FP1494001KT)	Akoya Biosciences, Marlborough, MA, USA	RTU	Opal 540
Secondary antibodies conjugated with horseradish peroxidase Opal 570 Reagent (#FP1488001KT)	Akoya Biosciences, Marlborough, MA, USA	RTU	Opal 570
Secondary antibodies conjugated with horseradish peroxidase Opal 620 Reagent (#FP1495001KT)	Akoya Biosciences, Marlborough, MA, USA	RTU	Opal 620
Secondary antibodies conjugated with horseradish peroxidase Opal 650 Reagent (#FP1496001KT)	Akoya Biosciences, Marlborough, MA, USA	RTU	Opal 650
Secondary antibodies conjugated with horseradish peroxidase Opal 690 Reagent (#FP1497001KT)	Akoya Biosciences, Marlborough, MA, USA	RTU	Opal 690
AmpliStain™ anti-Mouse 1-Step HRP (#AS-M1-HRP)	SDT GmbH, Baesweiler, Germany	RTU	HRP
AmpliStain™ anti-Rabbit 1-Step HRP (#AS-R1-HRP)	SDT GmbH, Baesweiler, Germany	RTU	HRP
4′,6-diamidino-2-phenylindole (DAPI, #D9542-5MG)	Sigma, Hamburg, Germany	5 µg/mL	w/o
VECTASHIELD^®®®®^ Mounting Medium (#H-1000)	Vector Laboratories, Burlingame, CA, USA	RTU	w/o
DAB Peroxidase Substrat Kit (#SK-4100)	Vector Laboratories, Burlingame, CA, USA	RTU	DAB
Toluidine blue (Biovitrum, #07-002)	ErgoProduction LLC, Saint Petersburg, Russia	RTU	w/o
Silver impregnation (Biovitrum, #21-026)	ErgoProduction LLC, Saint Petersburg, Russia	RTU	w/o
Picro Sirius Red Stain Kit (Connective Tissue Stain) (#ab150681)	AbCam, Cambridge, UK	RTU	w/o
Mayer’s Hematoxylin (Biovitrum, #05-002)	ErgoProduction LLC, Saint Petersburg, Russia	RTU	w/o

* RTU: ready to use.

**Table 3:** Designs of the diplex and multiplex immunohistochemical staining.

Design Challenge	Detection Targets	Label	Counterstaining of Nuclei
Immune landscape of the tumor microenvironment	Tryptase + CD3	Alexa Fluor 488 and Cy3	DAPI
	Tryptase + CD8	Alexa Fluor 488 and Cy3	
	Tryptase + CD14	Alexa Fluor 488 and Cy3	
	Tryptase + CD20	Alexa Fluor 488 and Cy3	
	Tryptase + CD38	Alexa Fluor 488 and Cy3	
	Tryptase + CD68	Alexa Fluor 488 and Cy3	
	Tryptase + CD138	Alexa Fluor 488 and Cy3	
	Tryptase + CD163	Alexa Fluor 488 and Cy3	
Immune landscape	Tryptase + CD3 + CD8 + CD14 + CD68 + CD163	OPAL 480, OPAL 540, OPAL 570, OPAL 620, OPAL 650, OPAL 690	DAPI (Akoya Biosciences, Marlborough, MA, USA)

## Data Availability

The original contributions presented in the study are included in the article/Supplementary Material; further inquiries can be directed to the corresponding author.

## Supplementary Materials

The following supporting information can be downloaded at: https://www.mdpi.com/article/10.3390/cells15010038/s1, Supplementary Figure S1: 3D model of targeted tryptase secretion to tissue and cellular targets. Supplementary Figure S2: 3D model of MC interaction with several endometrial stromal cells, including that through the formation of narrow cytoplasmic outgrowths. Supplementary Figure S3: 3D model of tryptase-positive cytoplasm, devoid of nucleus, with evidence of active tryptase secretion (arrowed). Supplementary Figure S4: 3D model of paracrine interaction between a MC and a killer T cell. Supplementary Figure S5: 3D model of juxtacrine and paracrine tryptase-mediated effects of MCs on cytotoxic T lymphocytes. Supplementary Figure S6: Juxtacrine colocalization of MCs and cytotoxic T lymphocyte forming a large contact area (arrowed). Supplementary Figure S7: Juxtacrine effects of two MCs on a killer T cell. 3D image. Supplementary Figure S8: Close MC localization with cytotoxic T lymphocyte. 3D image. Supplementary Figure S9: Juxtacrine (arrowed) and paracrine (double arrowed) effects of MCs on killer T cells. 3D image. Supplementary Figure S10: A small mast cell being in close contact with a CD14+ cell that has a prominent cytoplasmic extension. Supplementary Figure S11: MC interaction with multiple monocytes with targeted tryptase secretion. Supplementary Figure S12: Secretion of tryptase to a monocyte from a limited pole of a mast cell (arrowed). Supplementary Figure S13: Formation of cytoplasmic outgrowths of mast cell (arrowed), juxtacrine localization with monocyte. Supplementary Figure S14: Paracrine MC localization with CD14+ cell possessing an elongated outgrowth (arrowed). Supplementary Figure S15: Active tryptase secretion (arrowed). Supplementary Figure S16: Formation of a multicellular cluster of interacting CD14+ and MCs with active tryptase secretion (arrowed). Supplementary Figure S17: Close MC interaction with monocyte (arrowed). Supplementary Figure S18: MC interaction with CD14+ cell outgrowth. Supplementary Figure S19: Large area contact of a MC with a monocyte (arrowed). Supplementary Figure S20: MC embedding into the CD14+ endometrial cell network. Supplementary Figure S21: MC integration into the CD14+ endometrial cell network. Supplementary Figure S22: A MC contacting with several CD14+ endometrial cells. Supplementary Figure S23: Variant of MC interaction with the network of CD14+ endometrial cells. Supplementary Figure S24: Juxtacrine and paracrine MC interactions with type 1 macrophages. Supplementary Figure S25: Juxtacrine localization of a mast cell with high content of CD68 to type 1 macrophages. Supplementary Figure S26: A MC located at a paracrine distance to type 2 macrophage. Supplementary Figure S27: Volumetric image of juxtacrine interaction of a mast cell with type 2 macrophage. Large contact area. Supplementary Figure S28: Volumetric image of juxtacrine interaction of a mast cell with type 2 macrophage. Large contact area. Supplementary Figure S29: Representative isotype control images are presented for fluorescent microscopy (counterstaining wit DAPI) in (**A**), and for brightfield microscopy (counterstaining with hematoxylin) in (**B**) according to the procedure described in the sections “Methods”. Supplementary Figure S30: Summary box plot for mast cell (**A**) and CD8+ (*B*) density in the endometrium (cells per mm^2^). Notes: *, **, and *** represent *p* < 0.05, 0.01, and 0.001 levels of significance of difference, respectively. MiCE, mild chronic endometritis; MoCE, moderate chronic endometritis; and SCE, severe chronic endometritis.

## Abbreviations

MCs	mast cells
CE	chronic endometritis
AUB	abnormal uterine bleeding
RPL	recurrent pregnancy loss
PAR-2	Protease-Activated Receptor-2
