# Supplementary material for: Spatial Phenotype of the Mast Cell Population in Endometritis of Various Severities
Source: Cells. 2025 Dec 24;15(1):38. doi: 10.3390/cells15010038 (PMC12785079; doi:10.3390/cells15010038)
Supplement: Supplementary file 1 [file cells-15-00038-s001.zip › cells-3955274-supplementary.pdf]

Supplementary Figure S1

3D model of targeted tryptase secretion to tissue and cellular targets

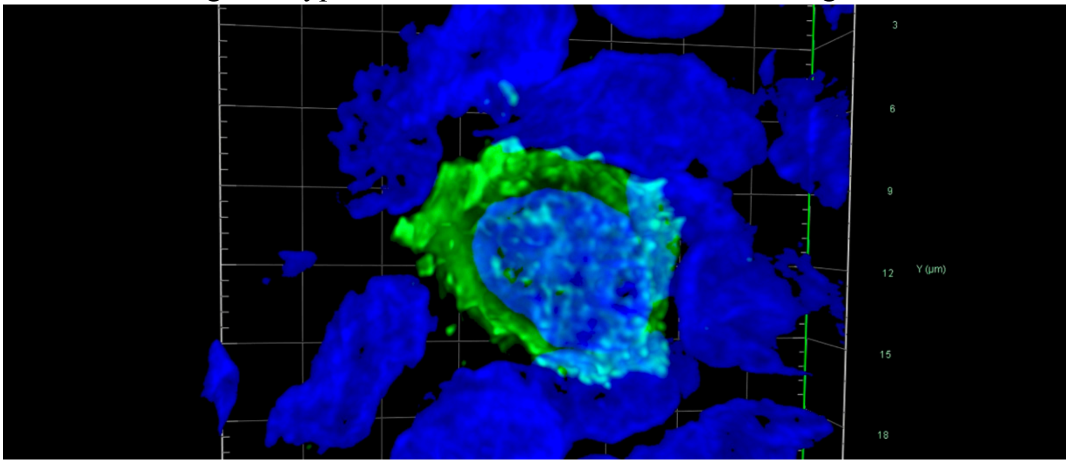

https://disk.yandex.ru/i/nyC2ubL0AmhEAQ

Supplementary Figure S2

3D model of MC interaction with several endometrial stromal cells, including that through the formation of narrow cytoplasmic outgrowths.

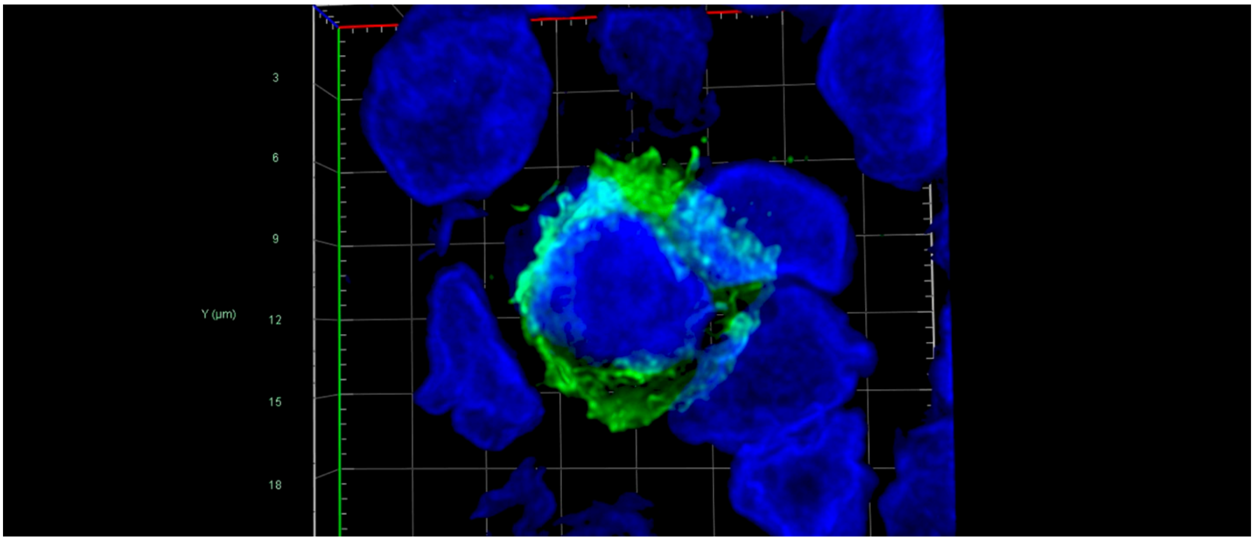

https://disk.yandex.ru/i/AKxSp74GWEJE5g

Supplementary Figure S3

3D model of tryptase-positive cytoplasm, devoid of nucleus, with evidence of active tryptase secretion (arrowed).

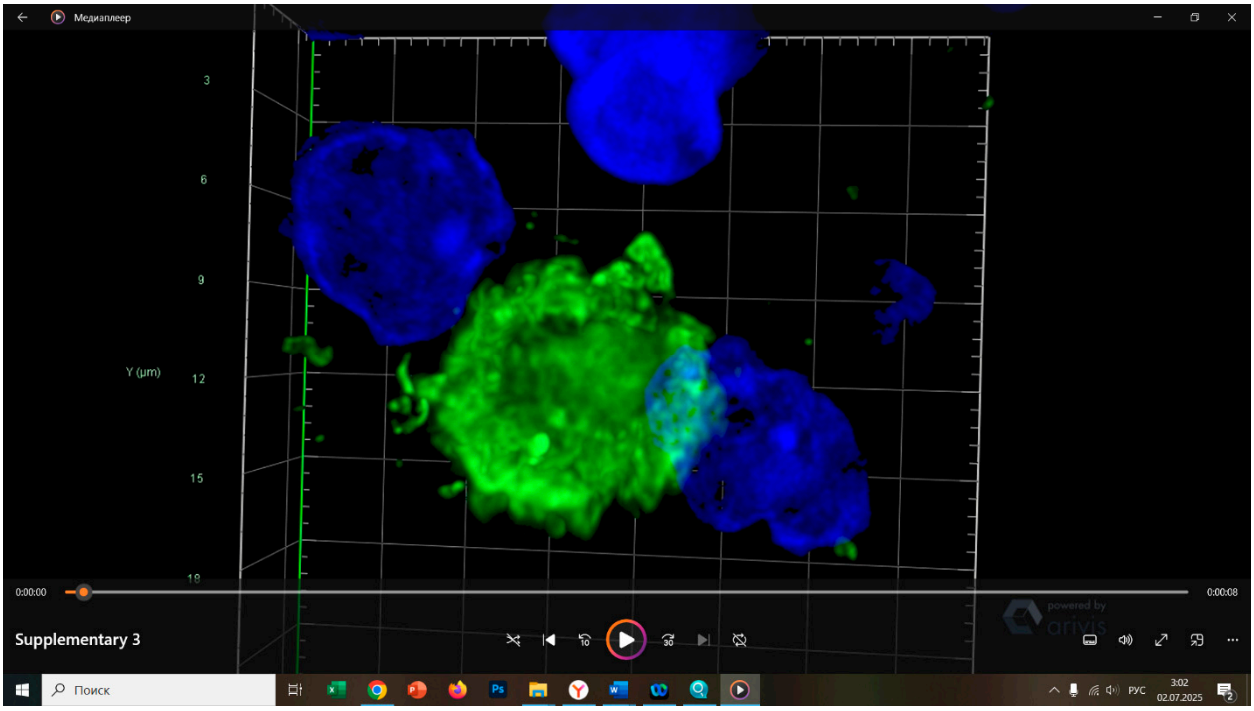

https://disk.yandex.ru/i/gbtynCx3PFTNiQ

Supplementary Figure S4

3D model of paracrine interaction between a MC and a killer T cell.

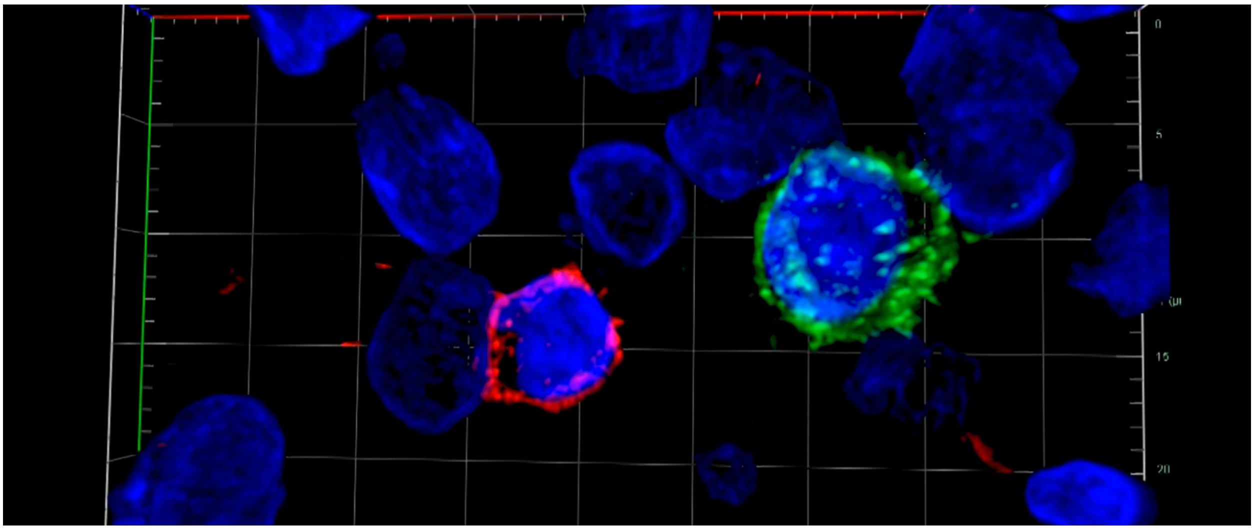

https://disk.yandex.ru/i/OS0WZ3fm4H74nQ

Supplementary Figure S5

3D model of juxtacrine and paracrine tryptase-mediated effects of MCs on cytotoxic T lymphocytes.

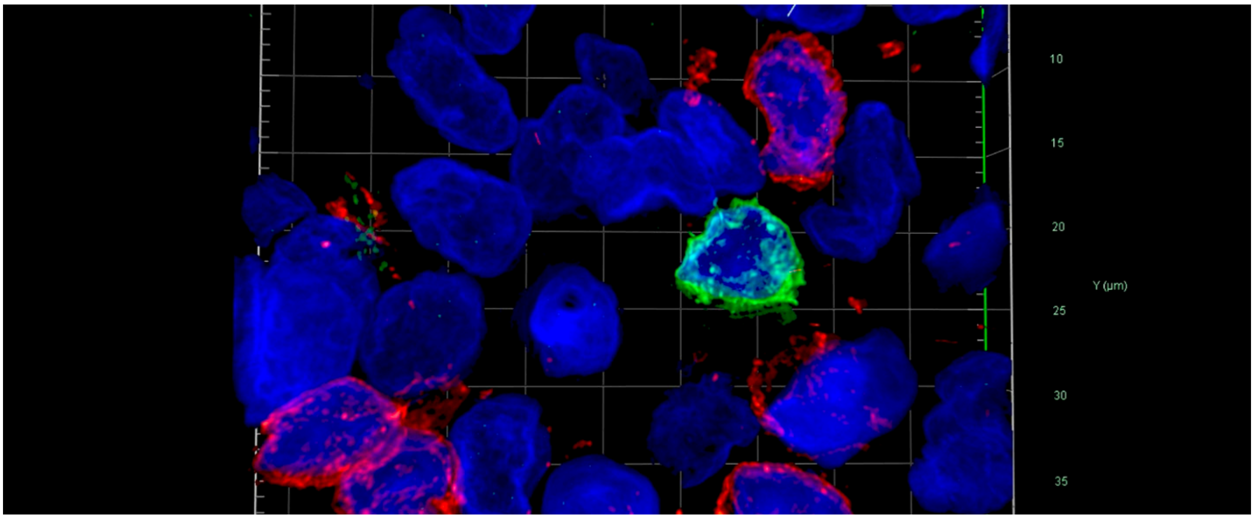

https://disk.yandex.ru/i/kW7Ax1vWAmd6bQ

Supplementary Figure S6

Juxtacrine colocalization of MCs and cytotoxic T lymphocyte forming a large contact area (arrowed). 3D model.

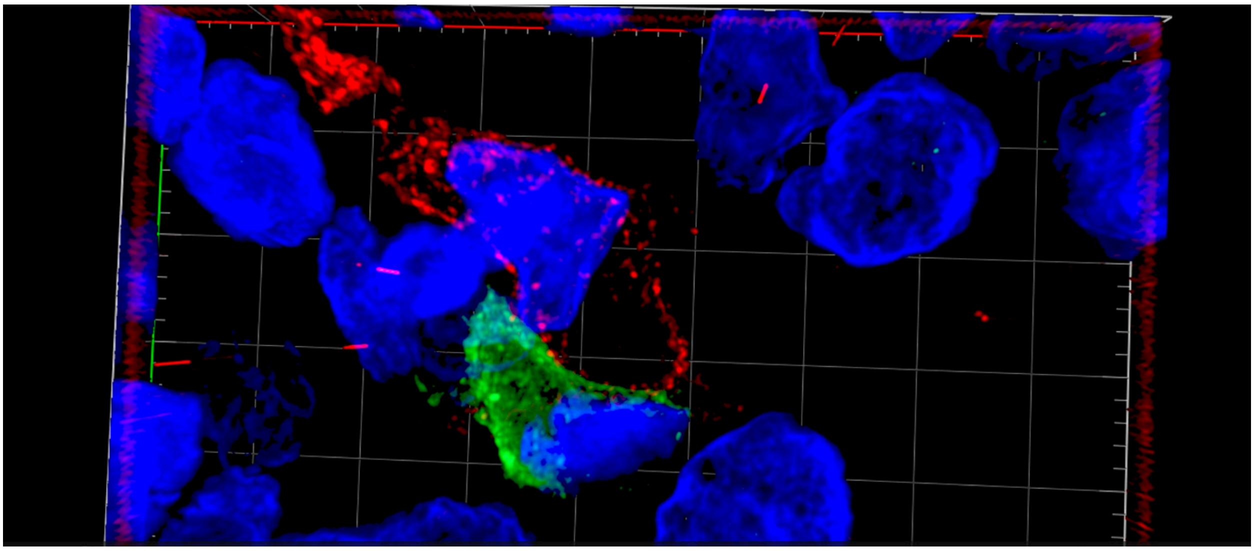

https://disk.yandex.ru/d/oprxSqNIw9sR7A

Supplementary Figure S7

Juxtacrine effects of two MCs on a killer T cell. 3D image.

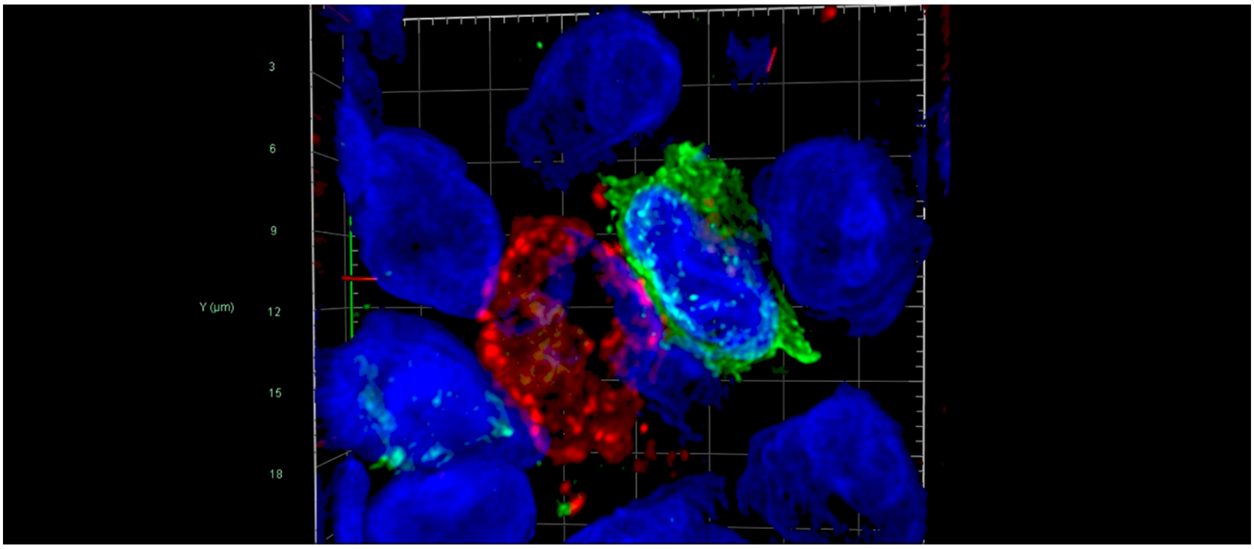

https://disk.yandex.ru/i/yj-88O5F4tX6kQ

Supplementary Figure S8

Close MC localization with cytotoxic T lymphocyte. 3D image.

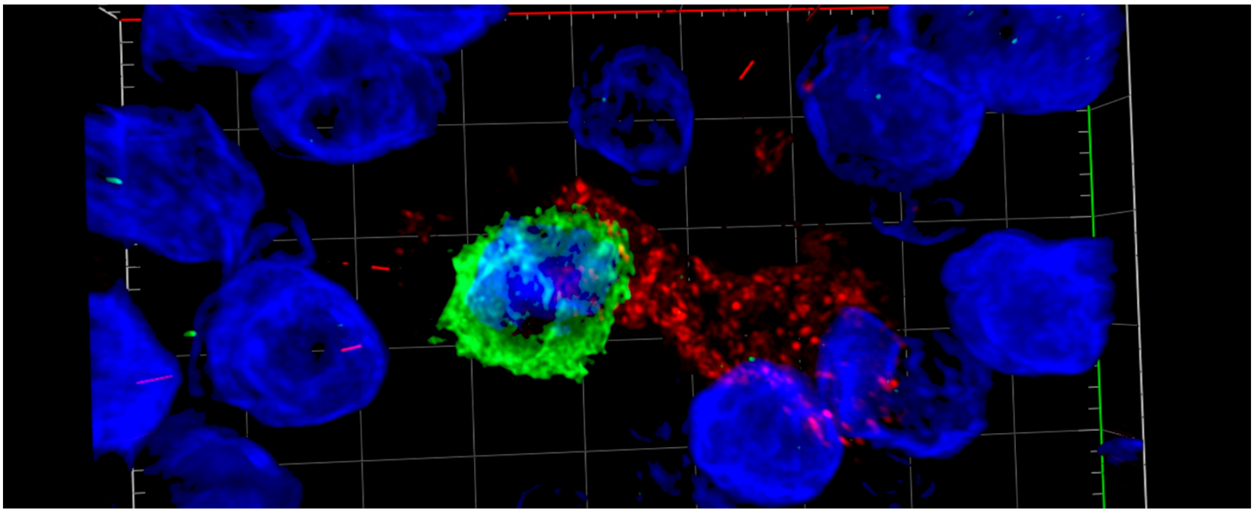

https://disk.yandex.ru/i/zQv8dkTpsZcoAg

Supplementary Figure S9

Juxtacrine (arrowed) and paracrine (double arrowed) effects of MCs on killer T cells. 3D image

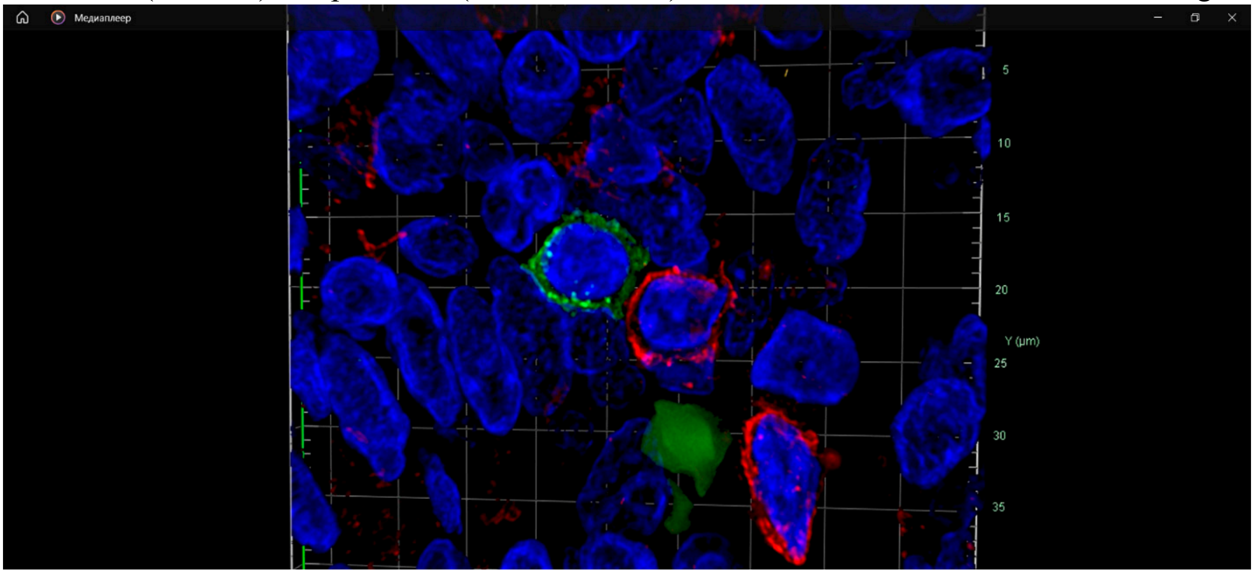

https://disk.yandex.ru/i/orBpRa8XxO0_7w

Supplementary Figure S10

A small mast cell being in close contact with a CD14+ cell that has a prominent cytoplasmic extension. 3D model

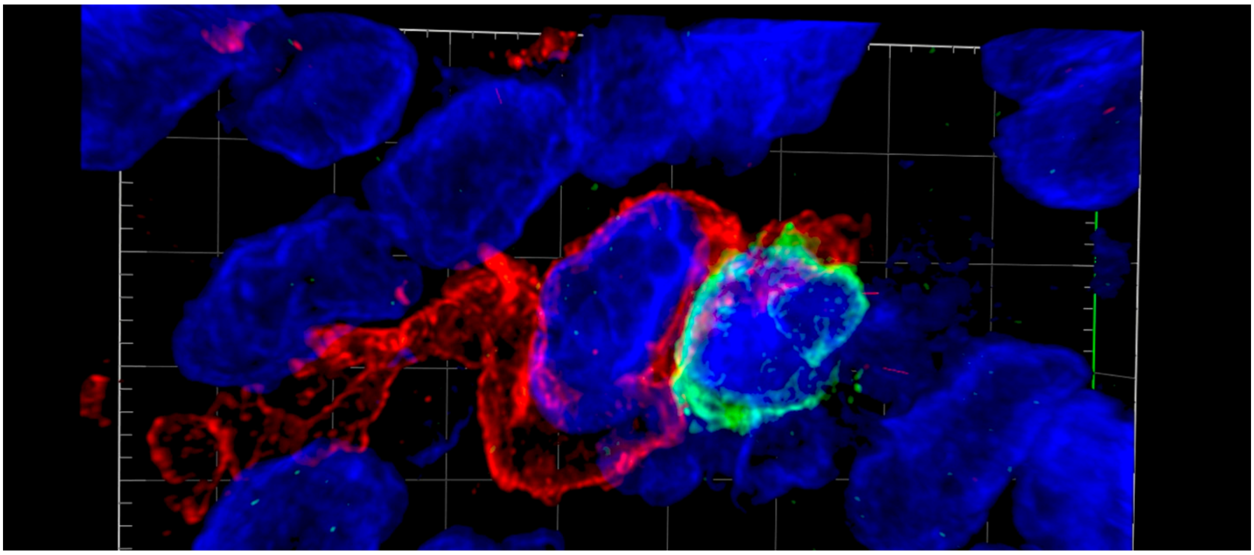

https://disk.yandex.ru/i/7ZKXSCI1WMjxPA

Supplementary Figure S11

MC interaction with multiple monocytes with targeted tryptase secretion. 3D model.

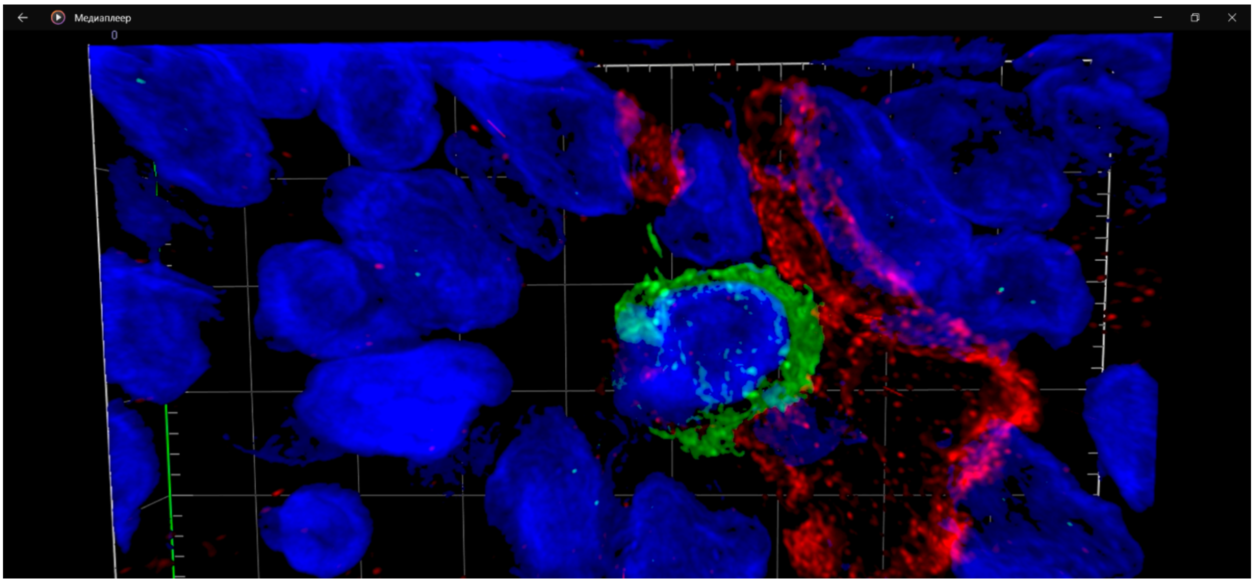

https://disk.yandex.ru/i/A9C5ARUW5leTjQ

Supplementary Figure S12

Secretion of tryptase to a monocyte from a limited pole of a mast cell (arrowed). 3D model.

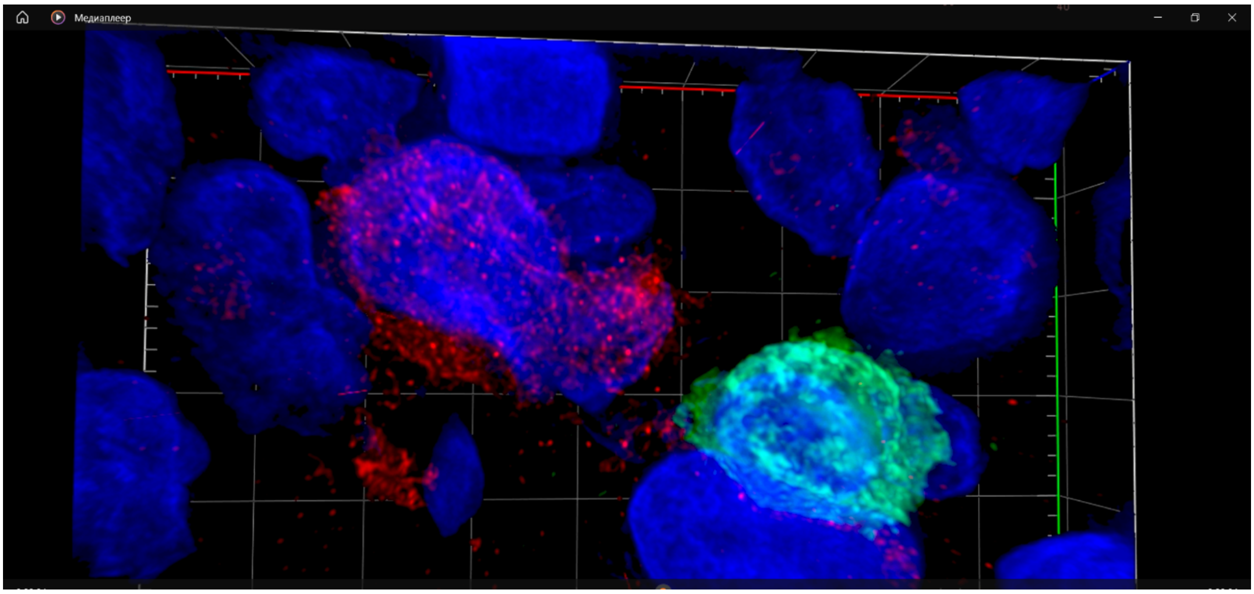

https://disk.yandex.ru/i/odVYtTobTsQqbA

Supplementary Figure S13

Formation of cytoplasmic outgrowths of mast cell (arrowed), juxtacrine localization with monocyte. 3D model.

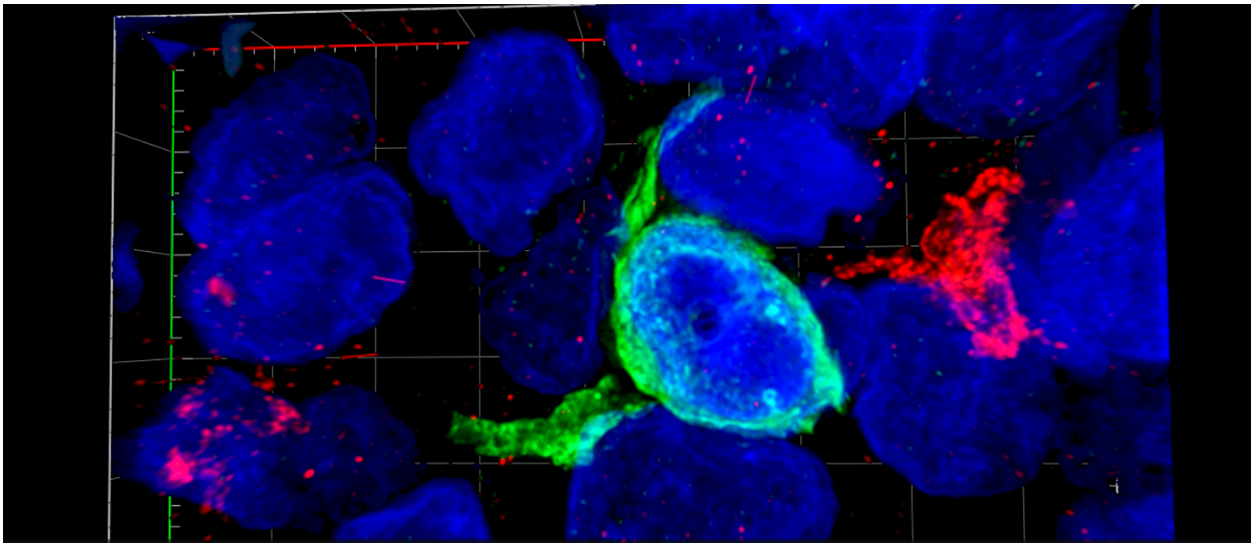

https://disk.yandex.ru/i/QyJqg9GzFR5MHA

Supplementary Figure S14

Paracrine MC localization with CD14+ cell possessing an elongated outgrowth (arrowed). 3D model.

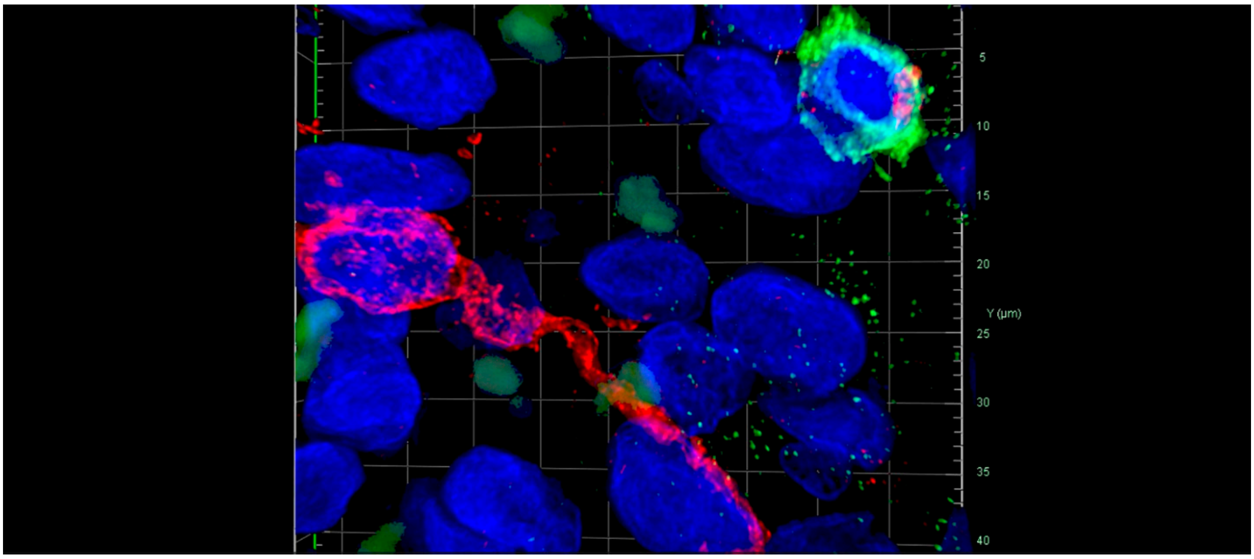

https://disk.yandex.ru/i/E5qszNR_9xp6Ng

Supplementary Figure S15

Active tryptase secretion (arrowed). 3D model.

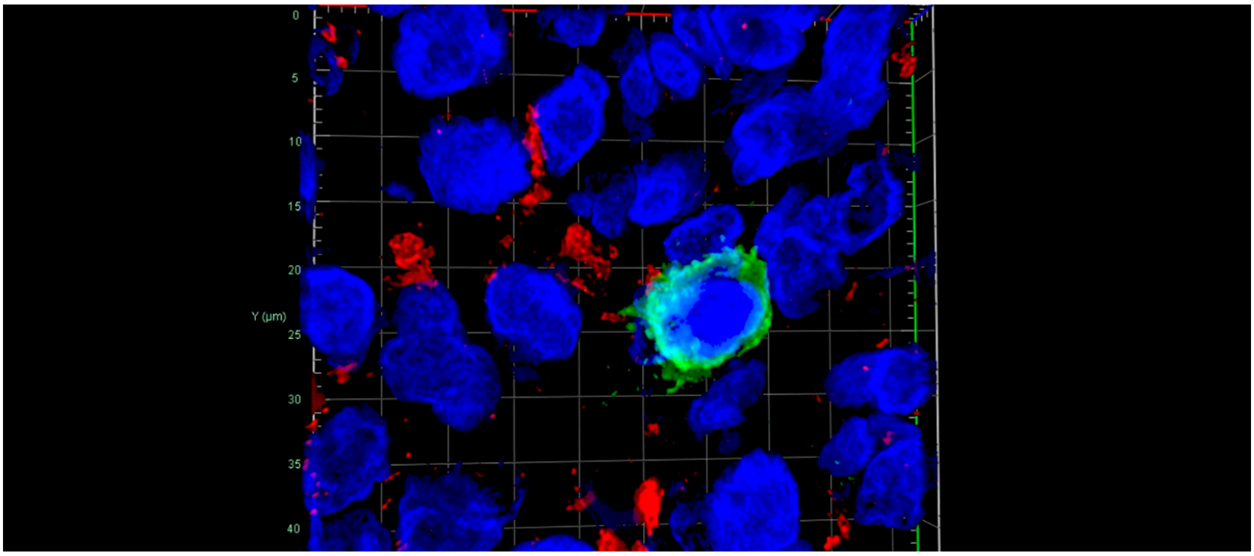

https://disk.yandex.ru/d/37b5nK-vQUAvaQ

Supplementary Figure S16

Formation of a multicellular cluster of interacting CD14+ and MCs with active tryptase secretion (arrowed). 3D model.

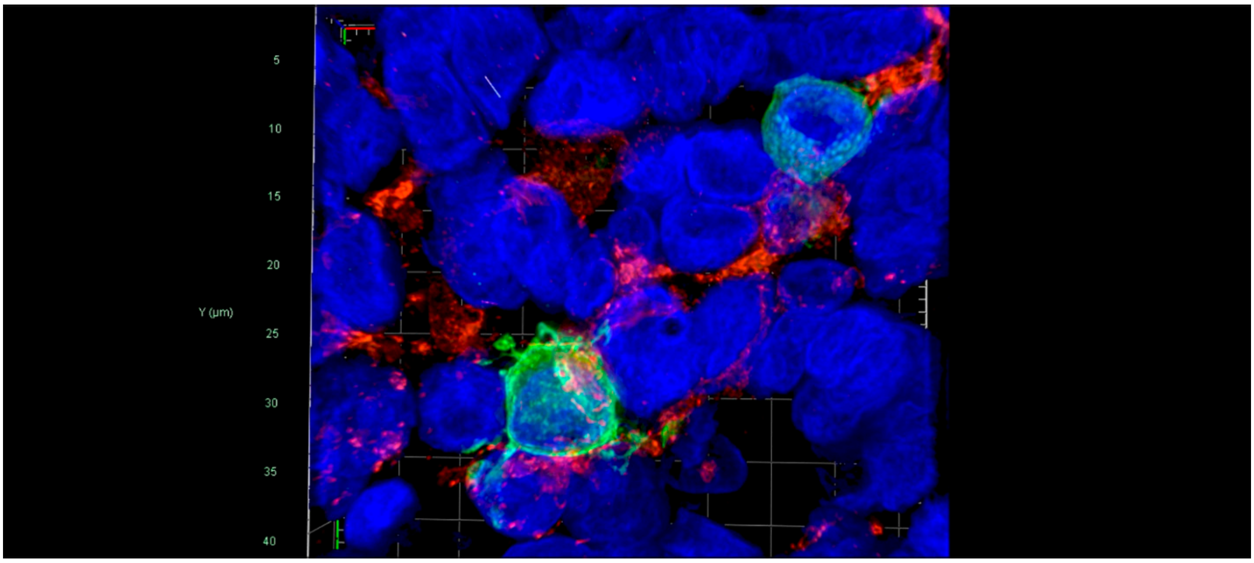

https://disk.yandex.ru/i/4dDBghnc2oeyog

Supplementary Figure S17

Close MC interaction with monocyte (arrowed). 3D model.

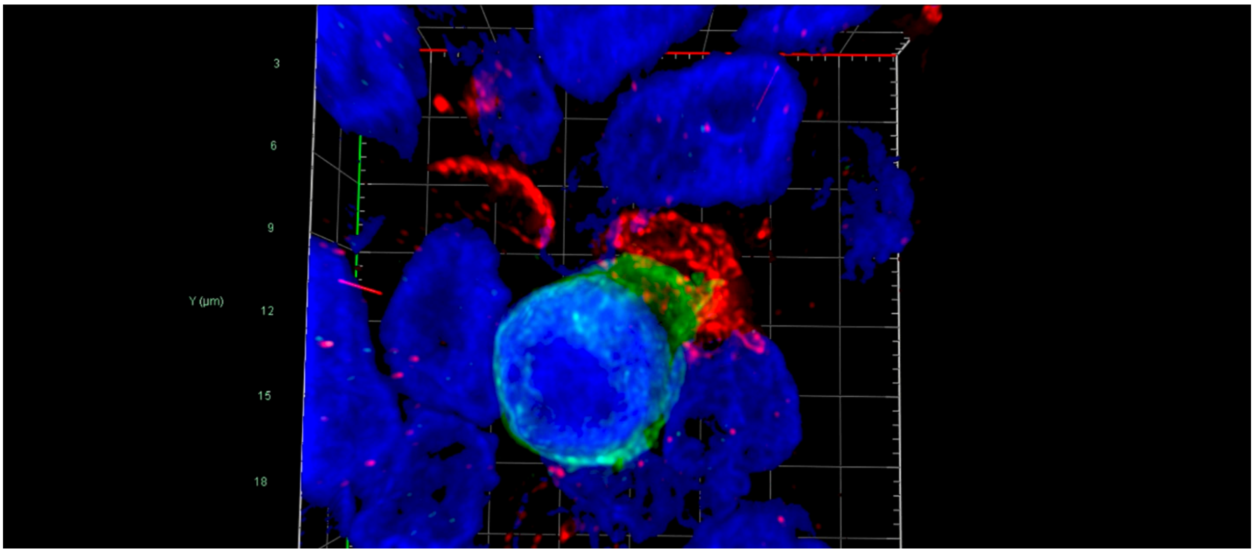

https://disk.yandex.ru/d/OQTA4Uz5tavYiA

Supplementary Figure S18

MC interaction with CD14+ cell outgrowth. 3D model.

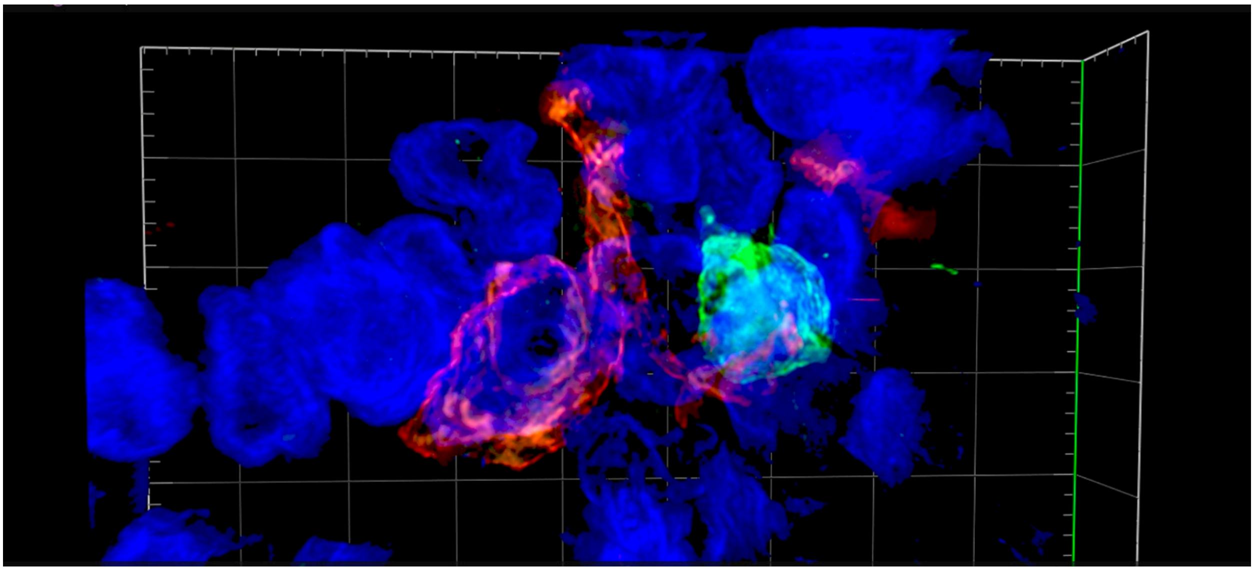

https://disk.yandex.ru/i/lKxfT9iwbEX9Qg

Supplementary Figure S19

Large area contact of a MC with a monocyte (arrowed). 3D model.

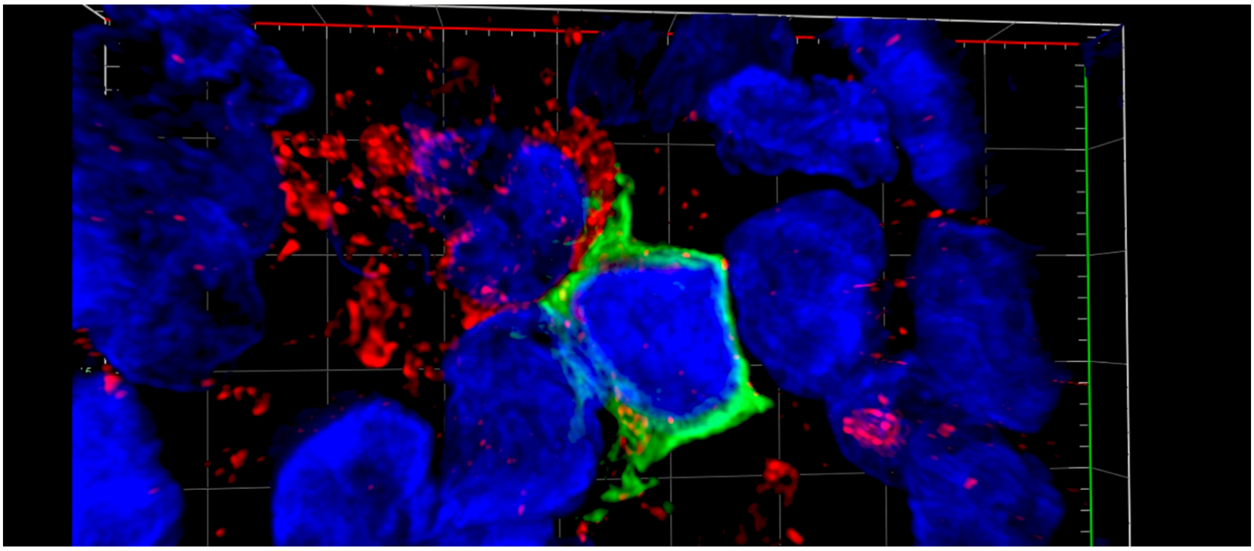

https://disk.yandex.ru/i/lG8OVAZgOV3ddg

Supplementary Figure S20

MC embedding into the CD14+ endometrial cell network. 3D model

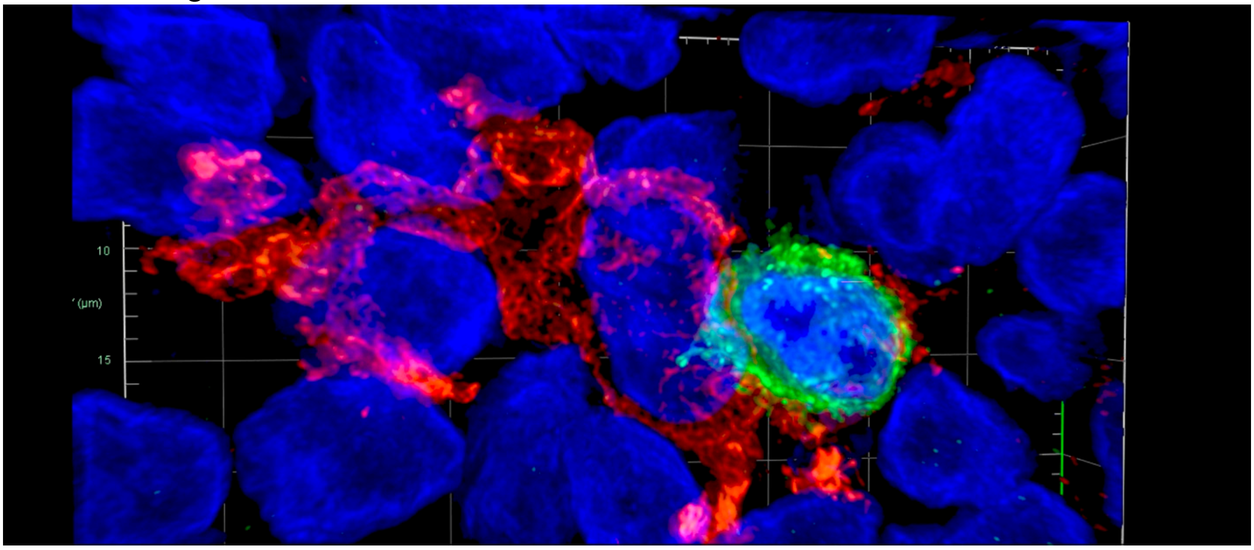

https://disk.yandex.ru/i/lthTIBnB9VCesw

Supplementary Figure S21

MC integration into the CD14+ endometrial cell network. 3D model

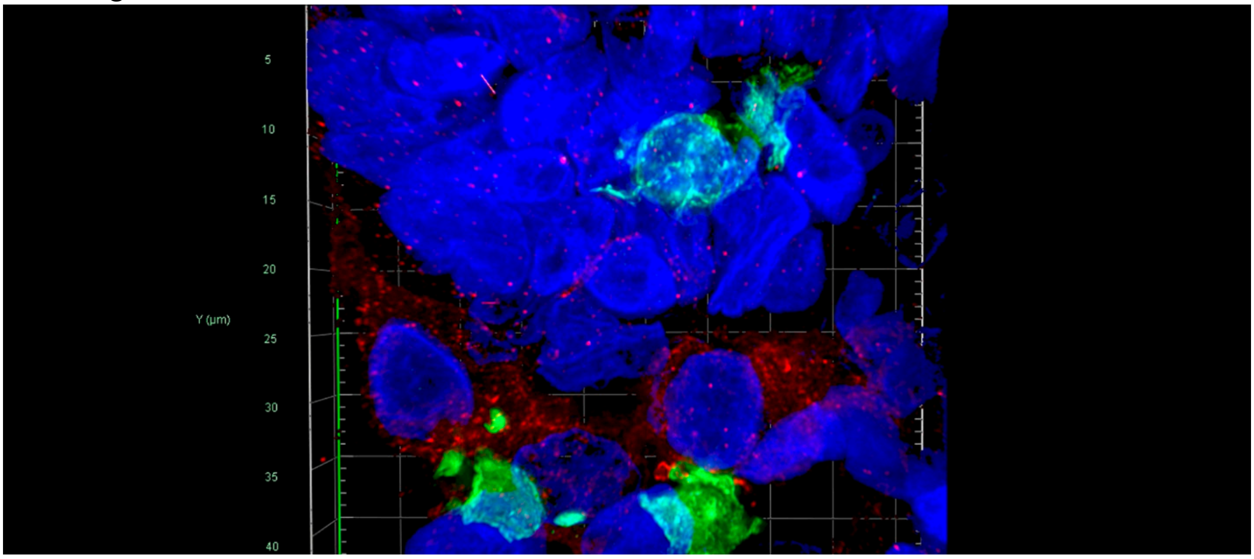

https://disk.yandex.ru/i/q5xTgw1IKJszSQ

Supplementary Figure S22

A MC contacting with several CD14+ endometrial cells. 3D model.

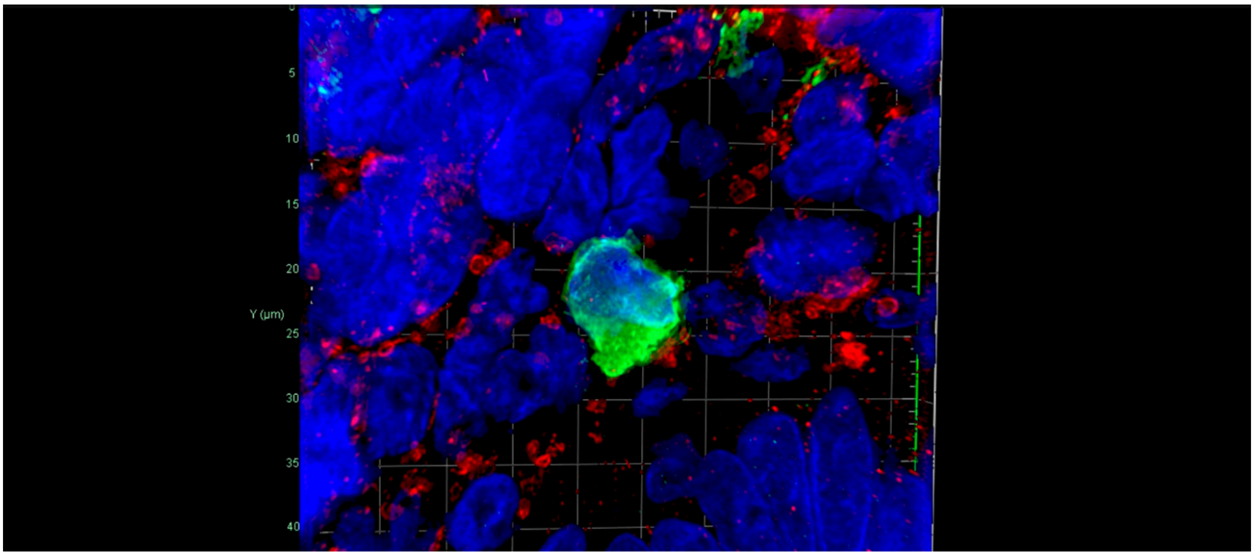

https://disk.yandex.ru/i/D9alWxcEtDdGYA

Supplementary Figure S23

Variant of MC interaction with the network of CD14+ endometrial cells. 3D model.

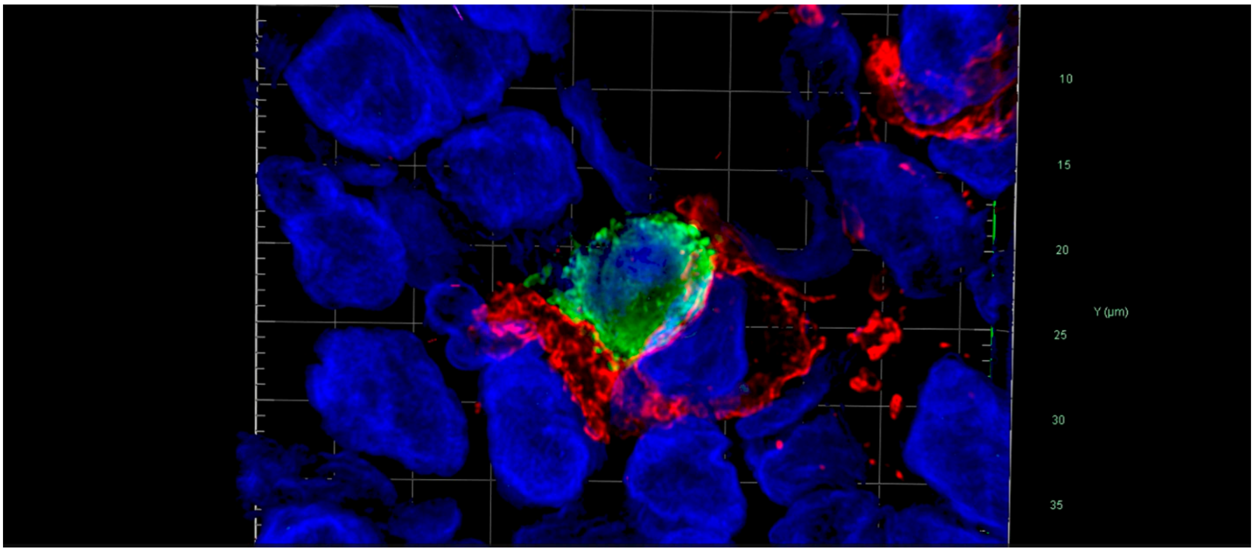

https://disk.yandex.ru/d/D2u5OKMjZAH67Q

Supplementary Figure S24

Juxtacrine and paracrine MC interactions with type 1 macrophages. 3D model.

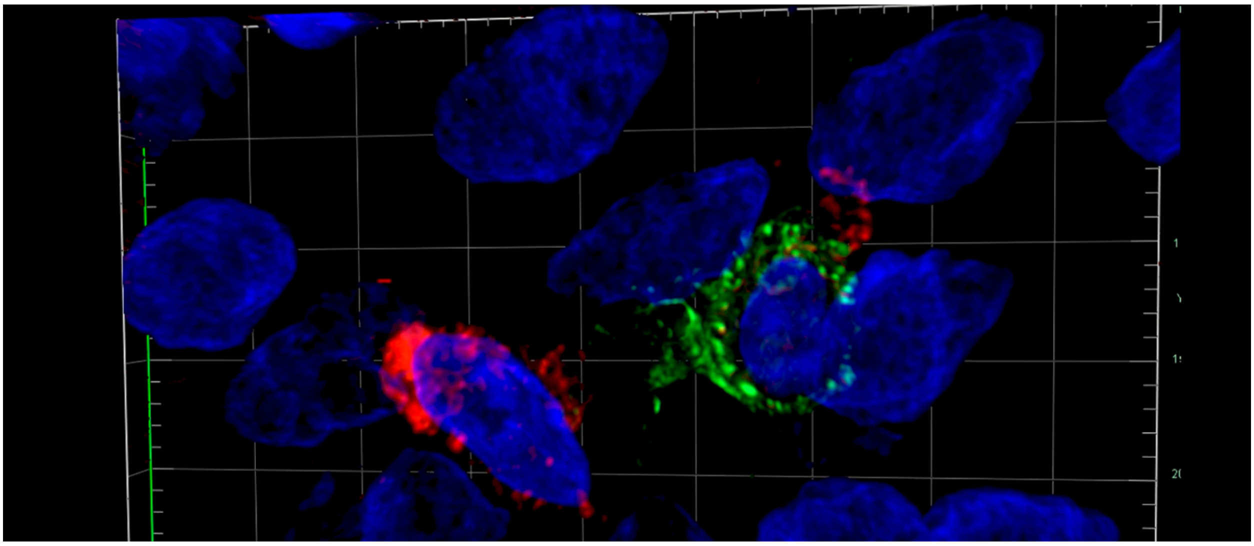

https://disk.yandex.ru/i/r_RcMrN6vUbPMA

Supplementary Figure S25

Juxtacrine localization of a mast cell with high content of CD68 to type 1 macrophages. 3D model.

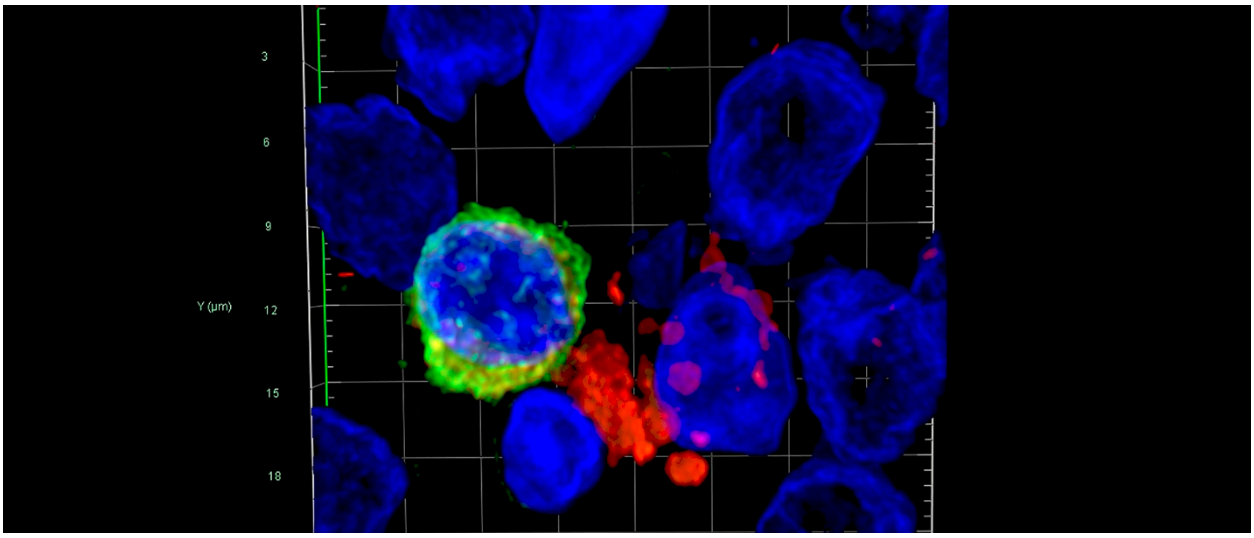

https://disk.yandex.ru/i/8UxAw7Pi_uVFPA

Supplementary Figure S26

A MC located at a paracrine distance to type 2 macrophage. 3D model.

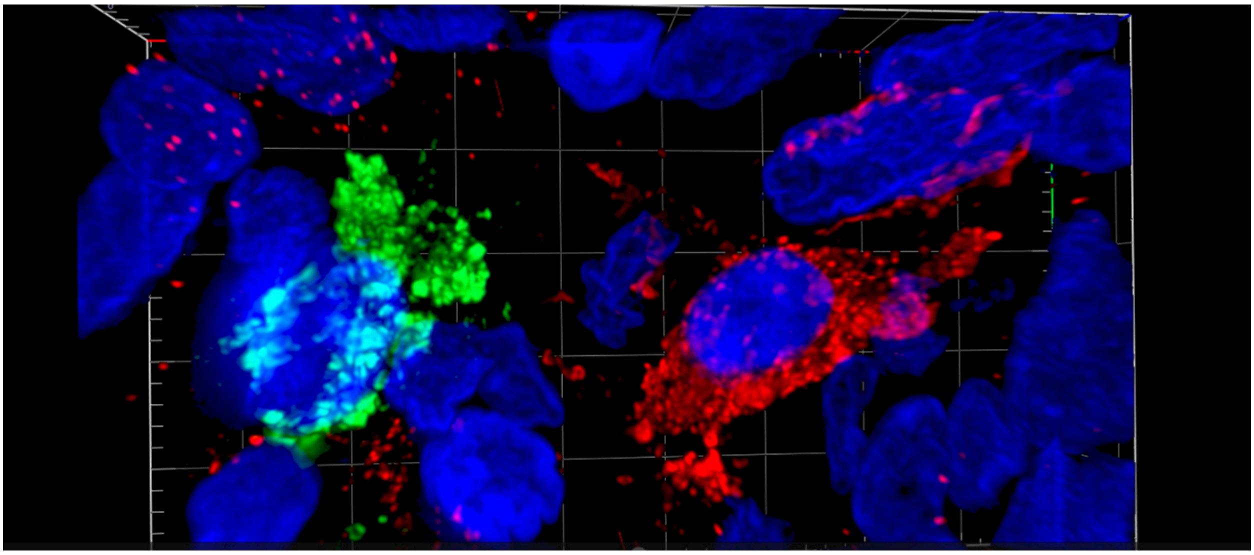

https://disk.yandex.ru/i/-eeGlqiDcBV-2A

Supplementary Figure S27

Volumetric image of juxtacrine interaction of a mast cell with type 2 macrophage. Large contact area. 3D model.

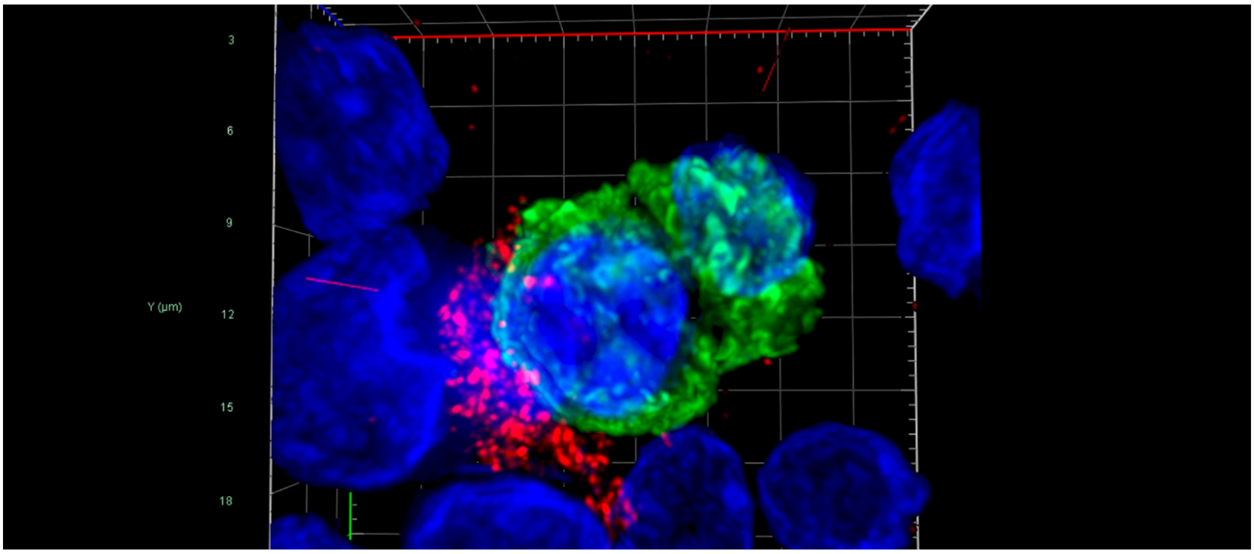

https://disk.yandex.ru/i/4jpDusquBsygHA

Supplementary Figure S28

Volumetric image of juxtacrine interaction of a mast cell with type 2 macrophage. Large contact area. 3D model.

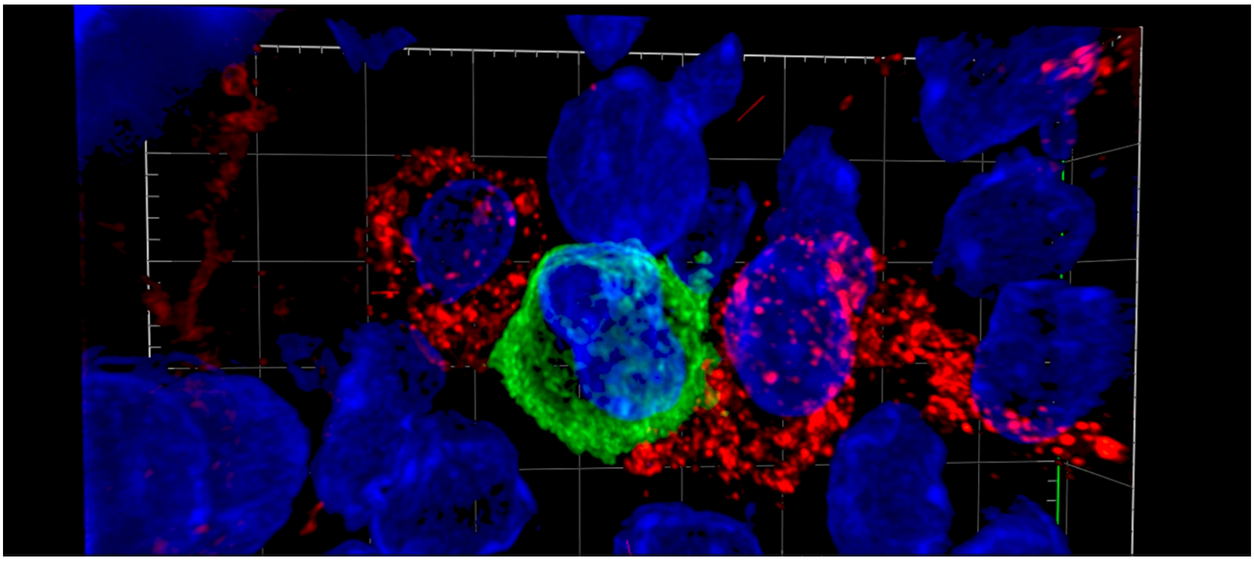

https://disk.yandex.ru/i/EfFrW8r_j_sBYQ

Supplementary Figure S29

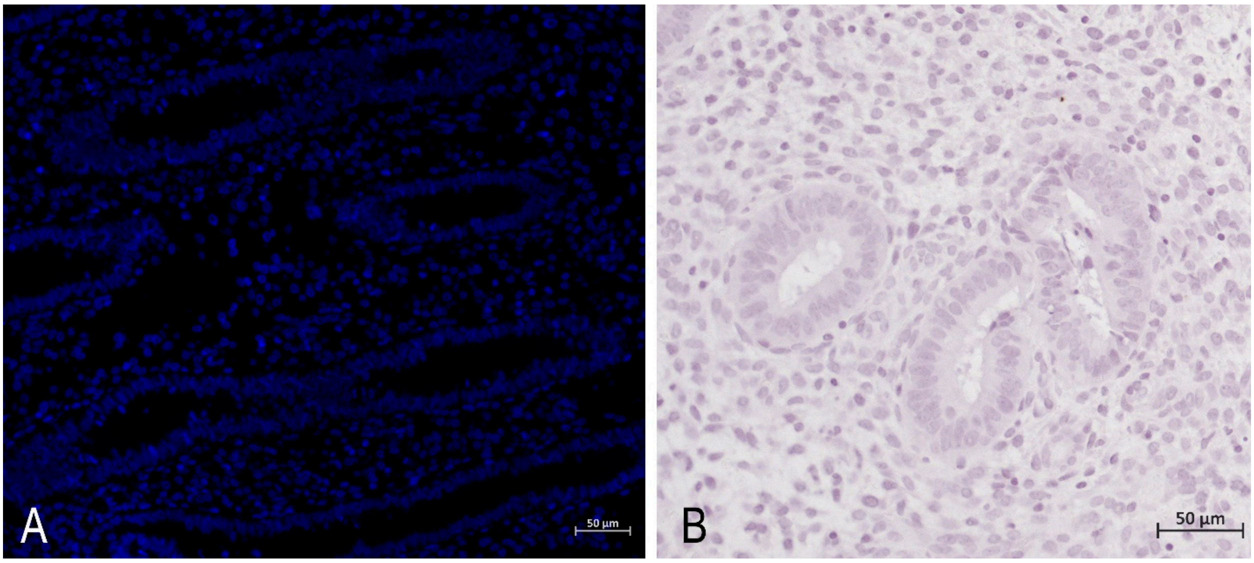

Representative isotype control images are presented for fluorescent microscopy (counterstaining wit DAPI) in (A), and for brightfield microscopy (counterstaining with hematoxylin) in (B) according to the procedure described in the sections “Methods”.

Supplementary Figure S30

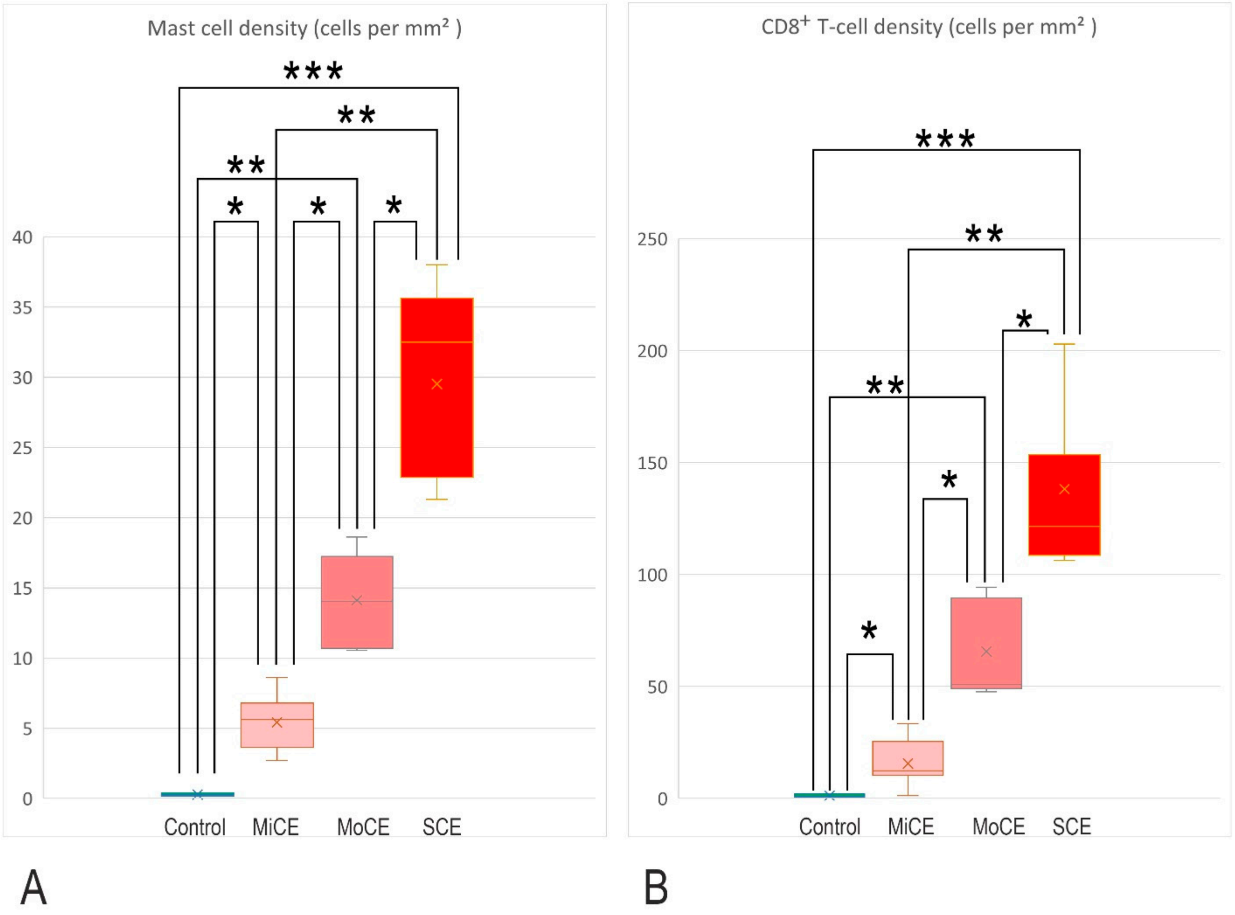

Summary box plot for mast cell (A) and CD8+ (B) density in the endometrium (cells per mm²). Notes: *, **, and *** represent p<0.05, 0.01, and 0.001 levels of significance of difference, respectively. MiCE, mild chronic endometritis; MoCE, moderate chronic endometritis; and SCE, severe chronic endometritis
